# Change point detection for clustered expression data

**DOI:** 10.1186/s12864-022-08680-9

**Published:** 2022-07-06

**Authors:** Miriam Sieg, Lina Katrin Sciesielski, Karin Michaela Kirschner, Jochen Kruppa

**Affiliations:** 1 Charité - Universitätsmedizin Berlin, corporate member of Freie Universität Berlin and Humboldt Universität zu Berlin, Institute of Biometry and Clinical Epidemiology, Charitéplatz 1, Berlin, 10117 Germany; 2 Charité - Universitätsmedizin Berlin, corporate member of Freie Universität Berlin and Humboldt Universität zu Berlin, Department of Neonatology, Charitéplatz 1, Berlin, 10117 Germany; 3 Charité - Universitätsmedizin Berlin, corporate member of Freie Universität Berlin and Humboldt Universität zu Berlin, Institute of Translational Physiology, Charitéplatz 1, Berlin, 10117 Germany; 4 Hochschule Osnabrück - University of Applied Sciences, Albrechtstr. 30, Osnabrück, 49076 Germany

**Keywords:** Simultaneous confidence intervals, Change point detection, Multiple contrast tests, Linear mixed models, Expression analysis

## Abstract

**Background:**

To detect changes in biological processes, samples are often studied at several time points. We examined expression data measured at different developmental stages, or more broadly, historical data. Hence, the main assumption of our proposed methodology was the independence between the examined samples over time. In addition, however, the examinations were clustered at each time point by measuring littermates from relatively few mother mice at each developmental stage. As each examination was lethal, we had an independent data structure over the entire history, but a dependent data structure at a particular time point. Over the course of these historical data, we wanted to identify abrupt changes in the parameter of interest - change points.

**Results:**

In this study, we demonstrated the application of generalized hypothesis testing using a linear mixed effects model as a possible method to detect change points. The coefficients from the linear mixed model were used in multiple contrast tests and the effect estimates were visualized with their respective simultaneous confidence intervals. The latter were used to determine the change point(s). In small simulation studies, we modelled different courses with abrupt changes and compared the influence of different contrast matrices. We found two contrasts, both capable of answering different research questions in change point detection: The Sequen contrast to detect individual change points and the McDermott contrast to find change points due to overall progression. We provide the R code for direct use with provided examples. The applicability of those tests for real experimental data was shown with in-vivo data from a preclinical study.

**Conclusion:**

Simultaneous confidence intervals estimated by multiple contrast tests using the model fit from a linear mixed model were capable to determine change points in clustered expression data. The confidence intervals directly delivered interpretable effect estimates representing the strength of the potential change point. Hence, scientists can define biologically relevant threshold of effect strength depending on their research question. We found two rarely used contrasts best fitted for detection of a possible change point: the Sequen and McDermott contrasts.

**Supplementary Information:**

The online version contains supplementary material available at (10.1186/s12864-022-08680-9).

## Background

Independent observations over time are counterintuitive. Examining samples at different time points, one would assume a dependent data structure between those. An ongoing aim of scientists is a better understanding of the underlying fundamental mechanisms that control organisms’ development. Scientists have investigated many genes, transcripts, proteins, etc. and their corresponding roles and have introduced models of connecting these networks. In our work, the observations between different time points were independent as the examination was lethal. The samples were measured at defined stages during gestation and later life (developmental stages) and were hence considered, in a broader sense, historical data. For reasons of reproducibility, more than one sample was measured at each time point and the measured parameter was gene expression. At each developmental stage, littermates and non-littermates were examined. Hence, we had a data setting with independent developmental stages but a dependent and independent data structure at each stage. The described setting is common for development studies in small mammals. Therefore, we want to present a novel methodology to find abrupt changes - so-called change points - in clustered historical gene expression data.

Two methodological approaches could be identified: A change point detection or a dose-response analysis. However, both ignore important aspects of our research question. A change point analysis assumes that the same subject is measured repeatedly over time and the data would therefore be dependent over time. Due to lethal examination of the mice, repeated measurement over time is not given in our data structure though. Therefore, change point detection algorithms assuming dependent points in time cannot be applied. Classical change point detection considered independent observations [1, 2] but is not easily accessible for the non-expert user.

The second methodological approach would be to analyze the developmental expression data with a dose-response analysis. In this setting, different increasing doses would be administered and the goal of the analysis would be to find the dose at which the (gene expression) response changes relevantly. As the measurement at each dose can be lethal, the observations are independent. In the dose-response setting, multiple contrast tests are widely used [3–5]. Nevertheless, there are differences to our biological setting, where the doses would correspond to developmental stages. Dose-response data is a type of progression. The developmental stages, however, do not lead to a monotonic increase in gene expression but can be up- and down-regulated over the time course. In a dose-response setting, a monotonic increase would be expected or an increase with an sudden decrease. In addition, the dose has defined units and therefore the distance between each dose should correspond to the change in dose. The developmental stage intervals in our example are not equidistant. It is, nevertheless, possible that the expression level of certain genes changes considerably during the lifetime of an individual. Changes could be due to maturation of certain organs or to birth [6–8]. The change could be gradual over time or very abrupt. In our work, such a developmental time point of an abrupt major change in gene expression is called a change point. Multiple contrast testing, e.g. using the Changepoint contrast, has been checked for statistical properties [9–11], but not been discussed for the purpose of detecting change points outside of the dose-response setting. Hothorn (2006) [12] shows the properties and a visualization of the Williams and Changepoint contrast in the setting of a randomized dose-response trials with a confidence intervaloriented approaches without clustering effects. Hothorn (2006) [12] also presents user specific contrasts, but which might be too complicated to build for non-expert users.

Moreover, not only the position of the change point (the corresponding developmental stage) was of interest but, for reasons of the underlying biological research question, also the corresponding effect size. In addition, we did not want to simply report the mean difference or the median difference, but also adjust the effect of the change point for possible confounders, which is not possible with classical machine learning methods for change point detection. In our view, the significance is not as important as the relevance [13]. Therefore, we focussed on the point estimator and the overall course of the confidence interval. This shift to informative effect estimates was required to make sure that findings can be reproduced on the way from basic research to clinical trials [14, 15]. Our approach using a log-normal transformation of measured expression values allowed estimating the effect of the change point. Depending on the measured parameter, linear mixed models could also be used to model the full range of the exponential family [11].

Therefore, we propose a novel application workflow to analyze historical data and return estimands for detected change points. In our case, we defined historical data as data consisting of a dependent structure between time points and a mixture of dependence and independence at each time point. We applied generalized hypothesis testing by using a linear mixed effect model as a possible change point detection method. We selected three potential contrast matrices for generalized hypothesis testing. When using a linear regression model, one can decide between effect parameterization and mean parameterization. In case of effect parameterization, one fits a model where the intercept is determined during the fitting process and all *β*-coefficients are dependent on and compared to the intercept. In case of mean parameterization, the intercept is set to zero and the calculated *β*-coefficients represent the mean of the corresponding variable. As we wanted to calculate the adjusted mean value for possible confounder effects for every time point, we decided to use mean parameterization. A linear mixed effect model with mean parameterization also allowed inclusion of the mix of dependent and independent data, while leaving the focus on the predictor of interest (the developmental time point). Generalized hypothesis testing offered the possibility to include multiple contrast scenarios. To our knowledge, this combination of methods has not been used to detect change points with an interpretable effect estimate. We tested the applicability of three established types of contrast matrices for our specific biological data setting. With two of those, we were able to obtain confounder adjusted effect estimates to detect change points. The effect at each potential change point could easily be interpreted by the non-expert user. Finally, we successfully applied the proposed method to in-vivo data presented in Kirschner et al. (2022) [16]

## Methods

In the following, we present a combination of model fitting and multiple contrast testing for the detection of change points in data which consisted of independent and dependent data points. However, dependence was not between data points at different but at the same time points and the observations were nested in each time point. As example, we used a developmental data set. The respective pups were nested through their mothers. At each time point, there were three new mother animals. Measurement of the expression levels is lethal for both, mother mice and their offspring. The aim was to find change points in historical gene expression data. In more detail, we wanted to find time points where the expression level of a gene majorly changed compared to the expression levels measured before, incorporating the underlying data characteristics. We tested our method on four biological sets of historical gene expression data and eleven simulated data sets. The simulation settings were designed by basic research scientists to ensure applicability. A flowchart of the main steps of the applied methods can be found in Supplementary Section 6 Fig. 17, Additional file 1.

### Biological expression data

We present a biological data set as a motivational example. In case of gene expression across developmental stages, e.g. in mice, the collection time points must be as few as possible but as many as necessary [17]. To assess relevant gene expression changes throughout the lifetime of relatively short-lived organisms like mice, one has to acquire data at specific, predefined time points during all developmental stages like embryonic, fetal, postnatal and adult. Predefined mouse development stages may be Theiler Stages (TS) and the day of birth (postnatal day: P) [18]. Data series in those cases consists of around 12-15 independent developmental stages. Additionally, at certain developmental stages and with certain data acquisition techniques, the examination is lethal and an individual can only be tested once. However, when lethal data acquisition is performed, ethical reasons demand examination of all pups in a litter [19]. To reduce the bias from one mother mouse and increase the sample size, pups from at least three mother mice are examined at each time point. The nesting leads to so-called mother effects and therefore dependency between certain data points. As each litter introduces its own variance, this information has to be taken into account when analyzing the data.

The expression data set is an extraction of a so-far unpublished study. We used the biological data as received (full course, not cleaned) to illustrate the proposed method. It is on the researcher to decide which developmental stages should be included depending on the research question. In detail, our example data consists of two genes in two mouse organs. We analyzed mouse livers and kidneys from thirteen developmental stages (embryonic to adult) for *glucose transporter 1 (Glut1)* and *carbonic anhydrase 9 (Car9)* expression by probe-based qPCR against a standard curve. The expression levels are displayed as *Glut1* or *Car9* molecules per 10^6^*βActin*(*Actb*) molecules. We used log-transformed expression values for our analysis to meet normality assumptions of the linear mixed model. We provide more information on the biological data in the Supplementary Section 2.1, Additional file 1. The four data sets were chosen because both genes showed a stable basal expression and a change of expression in only one of the organs. Expression changes from high-to-low (liver *Glut1*) and low-to-high (kidney *Car9*) were used to visualize our approach.

### Artificial expression data

**Fig. 1:**
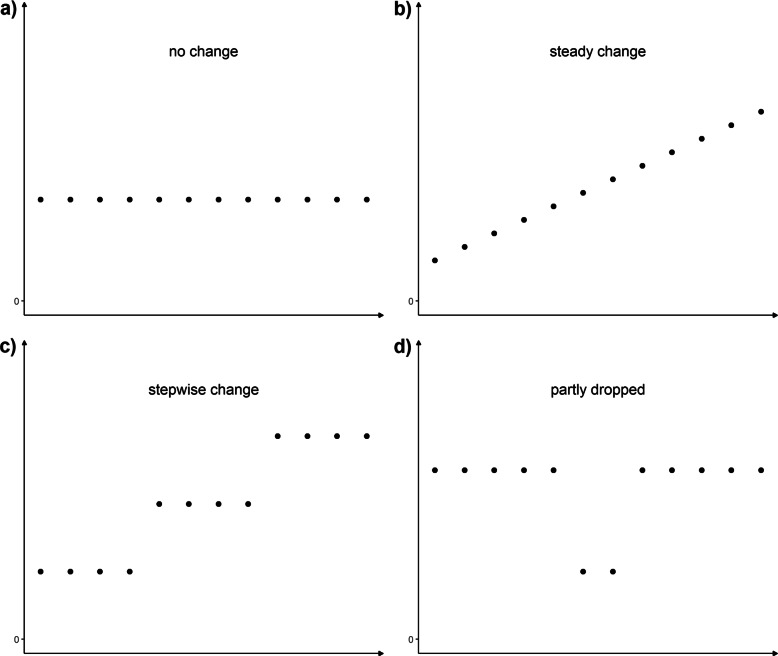
Possible courses of included historical data. Each subplot **a**) to **d**) represents one group of scenarios of courses within historical data. Points in time are on the x-axis, parameter values on the y-axis. Scenario **a**) shows a course with no change. Steady changes **b**) and stepwise changes **c**) each include increase and decrease of parameter values within the historical data. Scenario **d**) represents a partly dropped course which readjusts to previous parameter values after a while. The values may drop down to zero. These hypothetical time courses were provided. For scenarios **a**) and **b**), one would not expect any change points. In contrast, one would predict finding change points for scenarios **c**) and **d**). Example simulations for **c**) and **d**) can be found in Figs. 3 and 4

The researchers in the study defined four hypothetical historical gene expression data courses, representing biologically realistic and interesting scenarios. We simulated data with respect to the described data structure shown in Fig. 1. In detail, theoretical curves of the mean of the measured expression values for the respective time points in a time series were acquired. By the help of the theoretical courses, we were able to determine the properties of the different contrast tests. In total, four overall relevant courses of the means of the gene expression in the historical data were defined and were as follows: a) no change, b) steady change, c) stepwise change and d) partly dropped. In addition, we also simulated both directions (increase and decrease), if possible, simulating a linear increase as well as a linear decrease and so on.

We would not expect to detect change points in the historical data in scenarios a) and b). Therefore, both scenarios are our control or null models. However, for scenarios c) and d), we would expect detection of at least one change point. In addition, the confidence intervals should also provide more details on our findings. For each of the defined historical data scenarios, gene expression data for 12 distinct time points were simulated. As our biological example data had 13 developmental stages, we removed the adult stage to generate congruent data sets. The number has also good properties for the generation of the time points. For simulation of the expression data, we used the statistical programming language R 3.6 and the R package simstudy [20]. For each time point, we first generated three data points sampled from a normal distribution with a mean of zero and a variance of 5, the mother effects. These simulated mother values represented the individual effects each of the selected mother mice introduced on their respective litters. We did expect some mother effect, but no drastic differences at the same time point. We did choose a high mother variance, to achieve a more drastic setting. A very low variance would have generated very distinct expression values which we considered a very unrealistic setting. The amount of pups per litter was sampled from a Zero-truncated Poisson distribution with a lambda of 10. Therefore, each mother has an average of roughly 10 pups. The expression values of the mouse pups from the different litters were then generated by sampling from a normal distribution. The mean was based on the respective intercept and sampled mother effect. The variance was set to 2 since we expected only small differences between the expression values of the pups. We conducted a small simulation study for the variance of the mother effects with the values of 2, 6, and 10 and did not find any effect on the course of the confidence intervals showing that the linear mixed model was able to take into account the different mother variances. The simulation results can be found in the Supplementary Section 5, Additional file 1. In consequence, we had simulated expression values for pups from three different mothers for each of the 12 time points per defined course. For the more programming-oriented reader, we present the R code on a GitHub repository (https://github.com/msieg08/clustered_data_changepoint_detection) and code chunks in the Supplementary Section 4, Additional file 1.

We did not run different simulations with different sample sizes because the properties of the estimates from a linear mixed model in multiple contrast test is already well known. A general tutorial on linear mixed models using contrasts in R and the theoretical background can be found in Schad et al. (2020) [21], Bretz et al. (2016) [22] and Hothorn et al. (2008) [23]. Linear mixed models used in multiple contrast test will deliver unbiased estimates and will produce simultaneous confidence intervals on a 95% significance level. The properties are checked for heterogeneity [9], complex data models [10], and even under overdispersion and with small sample sizes [11]. Therefore, we consider the use of linear mixed models a valid and unbiased way to determine the estimates for the multiple contrast testing.

### Change point detection with linear mixed models and multiple contrast tests

To determine change points in our specific time series data, we first fitted a simple linear mixed effects model with mean parametrization. The expression data for one gene was set as the response. The different measurement time points were set as the fixed effects. The random effects part of the model were the mothers of the mouse pups. Therefore, the litter effect was accounted for and possible overdispersion was reduced. Equation 1 shows our simple linear mixed model with mean parameterization. For simplicity, only 5 (instead of 12) time points were illustrated.

1 $$ {}\overbrace{\mathbf{y}}^{150 \times 1}\ = \ \overbrace{\underbrace{\mathbf{X}}_{150 \times 5} \quad \underbrace{\boldsymbol{\beta}}_{5 \times 1}}^{150 \times 1} \ +\ \overbrace{\underbrace{\mathbf{Z}}_{150 \times 15} \quad \underbrace{\boldsymbol{u}}_{15 \times 1}}^{150 \times 1} \ + \ \overbrace{\boldsymbol{\varepsilon}}^{150 \times 1}   $$

with

$${}\mathbf{y} = \left[ \begin{array}{c} 26.45 \\ 23.71 \\ \vdots \\ 12.10 \end{array} \right] \quad \mathbf{X} = \left[ \begin{array}{ccccc} 1 & 0 & 0 & 0 & 0 \\ 1 & 0 & 0 & 0 & 0\\ \vdots & \vdots & \vdots & \vdots & \vdots \\ 0 & 0 & 0 & 0 & 1 \\ \end{array} \right] \quad \boldsymbol{\beta} = \left[ \begin{array}{c} 24.13 \\ 20.25 \\ 18.23 \\ 13.52 \\ 10.81 \end{array} \right] \begin{array}{l} t_{1} \\ t_{2} \\ t_{3} \\ t_{4} \\ t_{5} \end{array} \quad $$

$$\mathbf{Z} = \left[ \begin{array}{cccc} 1 & 0 &\ldots & 0 \\ 1 & 0 &\ldots & 0\\ \vdots & \vdots & \ddots & \vdots \\ 1 & 0 &\ldots & 1 \\ \end{array} \right] \quad \mathbf{u} = \left[ \begin{array}{c} 0.08 \\ 5.69 \\ \vdots \\ -4.71 \end{array} \right] $$

where

- **y** is the 150×1 vector of normally distributed expression values,
- **X** is the 150×5 design matrix for the fixed effects considering five time points (*t*_1_,...,*t*_5_),
- ***β*** is the 5×1 vector of the fixed effects coefficients due to mean parametrization the mean of each of the five time points (*t*_1_,...,*t*_5_),
- **Z** is the 150×15 design matrix for the random effects of the fifteen mothers with a constant intercept,
- **u** is the 15×1 vector of the random effects coefficients i.e. the effect of the mother on the expression with *u*∼*N*(0,5).

As a result, the *β*-coefficients represented the estimated mean values of the respective time points without the random effects variance introduced by the mothers. Using this approach, even more complex models with more confounders would be possible. In this study, we have concentrated on a simple model to illustrate the general framework. The effects of the time points could be adjusted as in any other multiple linear regression analysis. For further clarification, we provide a very short R code chunk as an example with the Changepoint contrast. The R terms can be matched to the formula 1 as follows. The expression indicates the **y**, the variable timepoint the **X*β*** as fixed effect, and the term (1 | mother) the **Zu** as random effects. The 1 in (1 | mother) indicates a constant intercept for all mothers. Mean parameterization was achieved by removing the intercept and placing 0 at the beginning of the lmer() formula. More complex code chunks are available in the Supplementary Section 4, Additional file 1. In addition, we provide further R code and functions on a connected GitHub repository (https://github.com/msieg08/clustered_data_changepoint_detection).

Therefore, we used the lme4 package [24] in R to fit the linear mixed models using the function lmer(). The function lmer() uses restricted maximum likelihood estimation by default to fit models that include varying random effects. The functionality determines the variances introduced by the random effects, here the mother effects. With respect to the variances, the rest of the model was fitted and the mean of each time point estimated. In the next step, change points were determined applying generalized linear hypotheses testing which utilized contrast matrices and directly performed multiple testing adjustment by applying a multivariate t-distribution. We tested different contrast matrices on the data to compare biologically relevant scenarios. In general, other endpoint distributions would be possible by modifying the proposed linear regression model. The function glmer() allows to fit the full range of the exponential distribution family. If required and with a sufficient sample size, one could add additional fixed or random effect variables like identifier of the PCR run or gender of the pups.

Tables 1, 2, and 3 show different contrast matrices. In the context of our work, the columns in a contrast matrix represent each existing time point and the rows represent possible scenarios. The scenarios can be considered as weighted comparisons between the time points. Each cell contains an assigned weight for the corresponding time point at the respective contrast. The sum of the weights equals zero for each row. There are different methods to calculate the respective weights depending on the type of a contrast matrix. In addition, each contrast could be adjusted for the number of samples per group i.e. unbalanced sample size. This latter adjustment is incorporated in the R package multcomp. In the context of this study, the following three types of contrast matrices were tested to detect change points: Changepoint, Sequen, and McDermott [25] from the R multcomp package [23]. Constructions of the contrast matrices to represent each of these types can be found in the Supplementary Section 4, Additional file 1.

**Table 1:** Changepoint contrast for five points in time and the resulting four contrasts. In C1 the first time point *t*_1_ is compared to the average of the other time points. In C2 the average of *t*_1_ and *t*_2_ is compared to the average of *t*_3_,*t*_4_, and *t*_5_

	t1	t2	t3	t4	t5
C 1	-1.00	0.25	0.25	0.25	0.25
C 2	-0.50	-0.50	0.33	0.33	0.33
C 3	-0.33	-0.33	-0.33	0.50	0.50
C 4	-0.25	-0.25	-0.25	-0.25	1.00

**Table 2:** Sequen contrast for five points in time and the resulting four contrasts. In C1 the first time point *t*_1_ is compared to the time point *t*_2_. In C2 the timepoint *t*_2_ is compared to *t*_3_ and so on. A zero indicates, that the time point is ignored for this specific contrast

	*t*_1_	*t*_2_	*t*_3_	*t*_4_	*t*_5_
1-2	-1.00	1.00	0.00	0.00	0.00
2-3	0.00	-1.00	1.00	0.00	0.00
3-4	0.00	0.00	-1.00	1.00	0.00
4-5	0.00	0.00	0.00	-1.00	1.00

**Table 3:** McDermott contrast for five points in time and the resulting four contrasts. In C1 the first time point *t*_1_ is compared to the second time point *t*_2_. In C2 the average of *t*_1_ and *t*_2_ is compared to *t*_3_. In comparison to the Sequen contrast the average of on increasing number of time points is compared to a single time point. Therefore, in the last contrast C5 the average of *t*_1_ to *t*_4_ is compared to *t*_5_

	t1	t2	t3	t4	t5
C 1	-1.00	1.00	0.00	0.00	0.00
C 2	-0.50	-0.50	1.00	0.00	0.00
C 3	-0.33	-0.33	-0.33	1.00	0.00
C 4	-0.25	-0.25	-0.25	-0.25	1.00

We constructed the contrast matrices in our study as follows: Each row of a contrast matrix consisted of one possible single change point scenario with respect to the selected construction method. Hence, the contrast matrix represents all possible single change point scenarios for the respective time series and selected method. Table 1 shows an example of the Changepoint contrast. If the Changepoint contrast is selected, the data is first divided into two groups for each row of a contrast matrix. One group contains the time points before the potential change point, the other group the time points at and after the potential change point. Then, the relative weight for each time point with respect to its group is calculated. Basically, the sample sizes from all time points of a group are summed and the sample size of each time point is divided by the respective sum. The sum of the weights from each group therefore adds up to one and the sum of the weights of both groups equals zero. The weights belonging to the time points before and at the possible change point are negated. If selecting the Sequen contrast, only the time point directly before and at the possible change point are considered. All other time points are set to 0. The time point directly before the possible change point is set to -1 and the possible change point is set to 1. Table 2 shows a numerical example.

Lastly, the McDermott contrast is a mixture between the Changepoint and the Sequen contrasts (numeric example Table 3). The weights of the time points of the time series before the possible change point are calculated the same way as for the Changepoint contrast. The sample sizes of each time point in this part of the time course are divided by summed sample sizes of this group. The possible change point itself is set to 1 and the rest of the time series is set to 0. The McDermott contrast matrix was originally invented for ordered means. A significant contrast in our setting would therefore suggest an overall significant change in the historical data, especially since our means are not ordered. In summary, Changepoint considers all data points in the time series, Sequen considers data points at and just preceding the potential change point, and McDermott only the data points at the time points before and at each potential change point.

How should we now match with our simulated settings (Fig. 1) with the contrasts? The Changepoint contrast compares the first time point with the mean of the following time points as well as the last time point with the average of the previous time points. It can be assumed that change points at the beginning and at the end would be more easily recognizable. By averaging the contrasts (C2 and C3 in Table 1) the discriminatory power between two neighboring time points decreases. The Changepoint contrast should therefore make it harder to separate the biological setting in Fig. 1c and d. The Sequen contrast always compares two adjacent groups or time points. Therefore, the Sequen may provide the exact point of change, but not the pattern of change. One could consider the Sequen as a repeated t-test - for two points at a time. The McDermott contrast, on the other hand, compares the current time point with the mean of all the previous time points. Thus, the McDermott contrast is able to show a progression. Both contrasts, Sequen and the McDermott, were able to show how the actual time point changed in contrast to the previous one and can correctly deptict the settings from Fig. 1c and d in the corresponding simulations in Figs. 3 and 4.

Taken together, we fitted linear mixed effect models for different biologically relevant time courses and for each of the four historical in-vivo gene expression data. To each fitted model, we applied three varying generalized hypotheses testing contrasts. The contrasts returned effect estimates for each scenario and respective 95% confidence intervals. The contrasts were evaluated on the basis of whether the respective contrast could be used to determine change points and whether it would potentially return the positions and directions of change points.

### Maximal number of usable steps

The presented approach has a theoretical limitation in the number of detectable significant differences. If many comparisons are included, each comparison will be corrected for the type I error. Therefore, at a given number of comparisons depending on the maximal observed effect size *δ*_*max*_ and the corresponding standard deviation *s*, no *significant* change point will be detected as significant. However, the point estimator of the confidence interval will not be influenced. In addition, the approximation also depends on the chosen contrast matrix. In the following, we will examine an approximation of how many comparisons can be analyzed. The scientist must estimate a *δ*_*max*_ and the corresponding *s* from the literature or the observed data. Then, we can calculate the *z*-score:

2 $$  z = \frac{\delta_{max}}{s}  $$

**Fig. 2:**
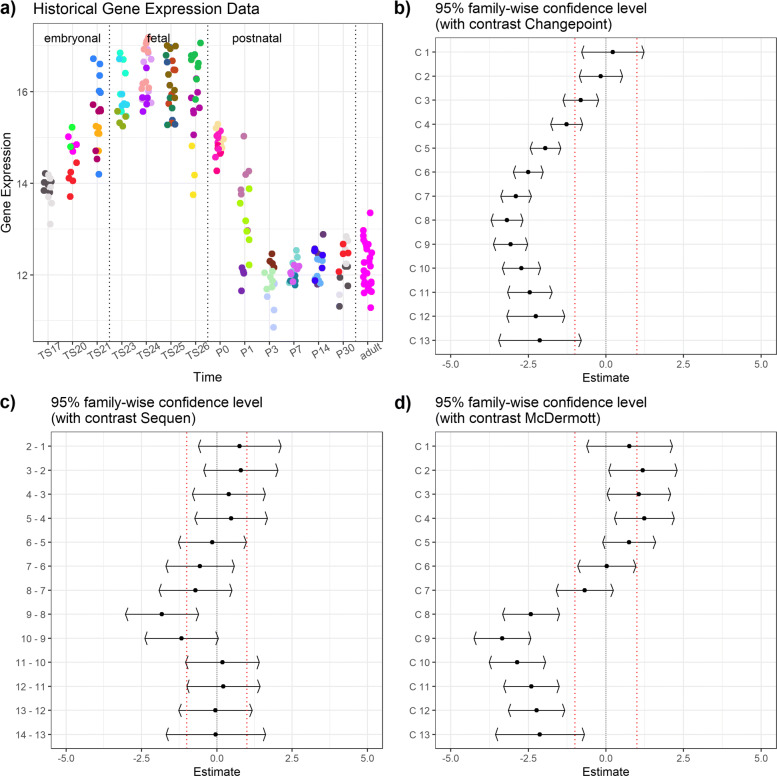
Biological example data for *Glut1* expression in the developing liver. Subplot **a**) shows the biological example data set (log-transformed). Each point in time on the x-axis represents an independent developmental stage. Each data point represents a pup and each color a mother animal. The pups are nested into the mothers. We added three broader development stages (embryonal, fetal, postnatal) for easier reference. The subplots show the confidence intervals of the Changepoint contrast (**b**), Sequen contrast (**c**), and McDermott contrast (**d**). The red scattered line indicates the chosen limits of biological relevance

The absolute value of the Z-score can be used by the probability density function of the normal distribution to calculate a *p*-value. In R, this can be achieved by the function pnorm(), which returns the integral from −*∞* to z of the probability density function of the normal distribution. Multiplying the result by two to account for a two-sided test resulting in the *p*_*max*_. and simplifying by assuming a Bonferroni adjustment, dividing 0.05 by *p*_*max*_ will determine the maximal number of theoretically possible detectable significant change points. The emphasis is on theoretical, because if we are not able to find any significant *p*-value, we will also not find any significant confidence intervals.This is only an approximation, please be referred to the discussion section for further considerations. A small numeric example is given in Fig. 2a showing a *δ*_*max*_ of 3 between the two plateaus. Assuming a standard deviation of 1, we can calculate a z of $\tfrac {3}{1}$ equal 3. Using the function pnorm(-3) we get a *p*-value of 0.00135. Hence, we would be able to run approximately 37 comparisons in our analysis with at least one significant confidence interval but we recommend not to concentrate on the significance but to rather consider the course of the point estimators. Since the confidence intervals directly represent the effect estimator, the user must decide whether the change point is relevant for the biological question. The confidence intervals provide a measure for the uncertainty, but the number of comparisons is also integrated by the width of the confidence intervals.

## Results

The following section is divided into two parts. First, we present four motivational biological data examples, three of which can be found in the Supplementary Section 2, Additional file 1. The mouse development data set underlines the biological necessity of our approach. Second, we simulate different course settings inspired by the biological data. We show the resulting confidence interval plots for each simulation and contrast and separately report the effect estimates.

In all presented plots, subplot a) shows the respective data with time points on the x-axis and the measured expression values on the y-axis which we assume to have at least a log-normal distribution. Each dot in the plot represents one measured value. The colors represent the data dependencies, meaning that dots with the same color belong to the same cluster, e.g. pups from the same mother. Subplots b) to d) show the estimated mean difference including the 95%-confidence interval (x-axis) for each respective change point scenario (y-axis).

### Biological gene expression data

**Fig. 3:**
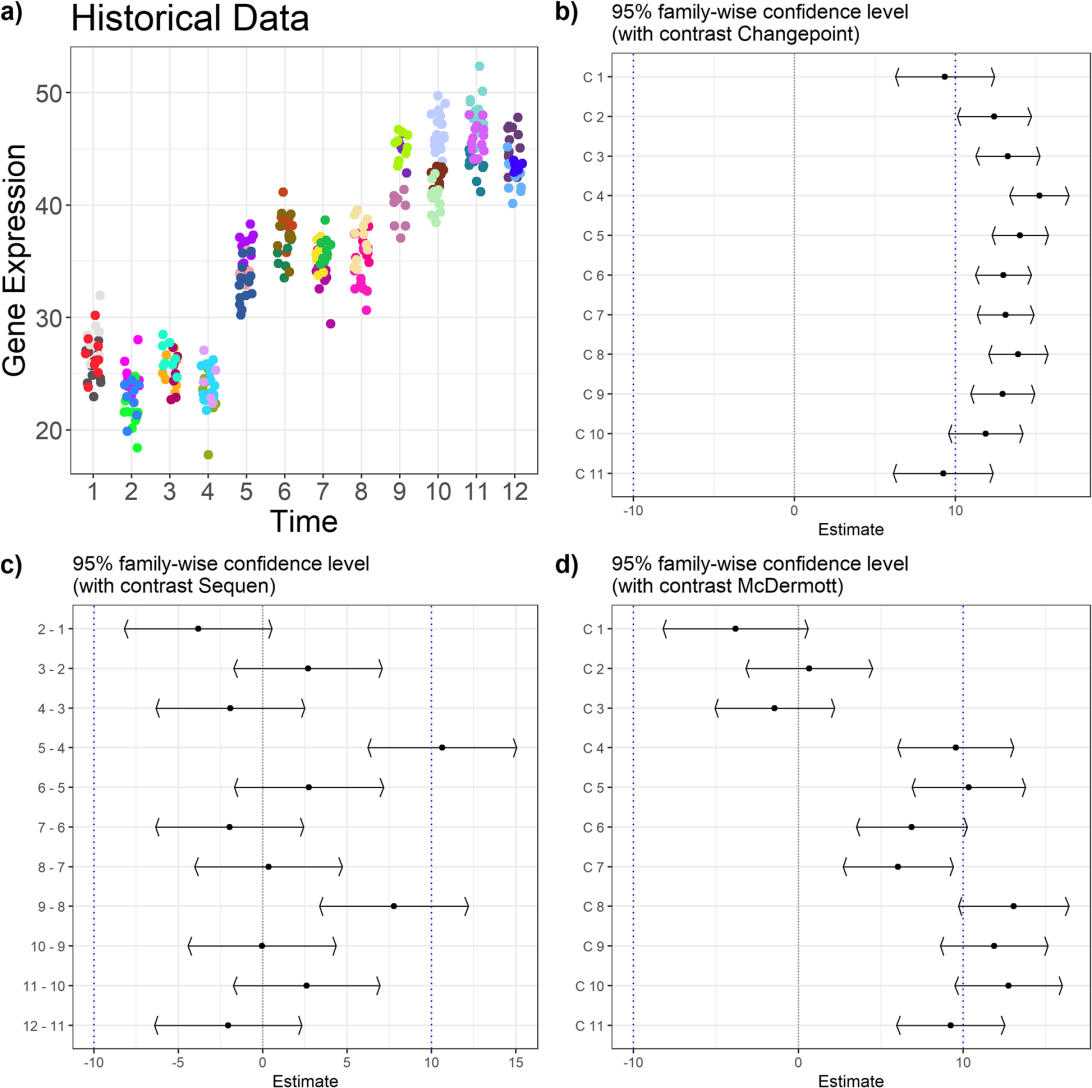
Confidence intervals of estimates from linear mixed model coupled with contrast matrix for historical data with two change points. Figure **a**) shows the points in time (x-axis) of the sampled historical data in association with gene expression (y-axis) with two expected change points. Each color is related to one mother mouse. Subfigures **b**), **c**) and **d**) show the estimates (x-axis) including confidence intervals for the observed contrasts (y-axis) with methods Changepoint, Sequen and McDermott, respectively. The blue line indicates the simulated effect

Our motivational biological example data include the developmental *Glut1* gene expression in the liver (Fig. 2, numerical effect estimates in Table 4) and kidney (Supplementary Section 2, Additional file 1), respectively. The estimation of the model parameters of the *Car9* expression during kidney development (Supplementary Section 2 Fig. 1, Additional file 1) caused converting problems as we observed singular fits. This was not the case for *Car9* expression data from developing liver (Supplementary Section 2 Fig. 2, Additional file 1) or *Glut1* expression data from kidney (Supplementary Section 2 Fig. 3, Additional file 1). All plots have the same structure and consist of the same subplots. The subplot a) shows the biological data separated into three developmental stages. Each dot represents a single pup nested into a single mother which is indicated by the same (litter) color. Please note that the expression data is log-transformed. The other subplots show the results of the different contrast tests: b) Changepoint, c) Sequen, and d) McDermott. The scattered line indicates the biological relevance limits. These limits are user-specific and depend on the research question. We chose ± 1 for our example.

**Table 4:**
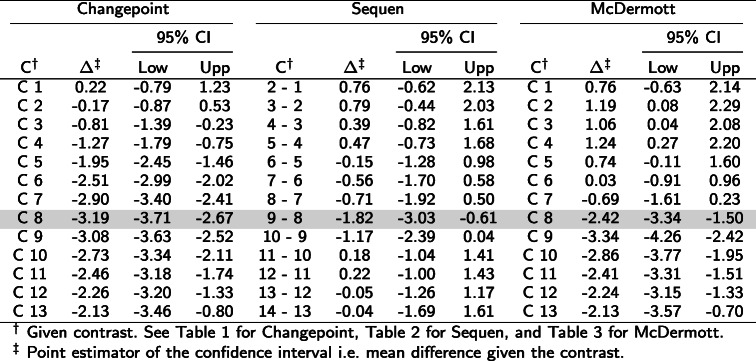
Contrasts and estimates of Fig. 2. The table shows the numeric values for the *Glut1* example data from liver. The C column indicates the contrast, the *Δ* the log mean change of the corresponding contrast C. The gray row indicates a possible change point by visual inspection of Fig. 2. A significant confidence interval does not include zero

^*†*^Given contrast. See Table 1 for Changepoint, Table 2 for Sequen, and Table 3 for McDermott.

^*‡*^Point estimator of the confidence interval i.e. mean difference given the contrast

Figure 2 shows an example of a visually obvious change point with severe expression changes after birth (from P0). This change point is indicated by a gray line in Table 4. The Changepoint contrast visualized the overall course of the time points more than the rapid decrease from TS26 to P3 and it did not deliver a clearly interpretable position of the change. The averaging over all time points concealed the linear increase between the TS17 and TS21 developmental stages because the decrease at the end of the time points is too severe. In contrast, the Sequen contrast detects the change point at the 9-8 position (P0-P1) with an effect of -1.82 [-3.03; -0.61]. Due to the mixed modeling, we were able to account for the high variance of developmental stage P1. However, no confidence interval fell below the lower relevance limit. The McDermott contrast showed confidence intervals below the relevance limit with an effect of -2.42 [-3.34; -1.50] at birth. In the following, the confidence intervals had a point estimate around -3.2. The slight increase in the beginning was also pictured in the course of the confidence intervals with an effect around 1.

Supplementary Section 2 Fig. 1, Additional file 1, shows the biological data of the *Car9* gene from kidney (numerical values in Supplementary Section 2 Table 1, Additional file 1). The estimation of the model parameters caused converting problems. We achieved singular fits, therefore got estimated variance-covariance matrices with less than full rank. The warning indicated that one or more variances were very close to zero. Therefore, a careful consideration of the results is required. We are sure to avoid the fitting of overly complex models [26] and assured consistency of the model with the experimental design [27]. Therefore, we believe that the mean estimates and the variance /covariance matrices were valid, even if mixed models can show converting problems. The biological data showed a plateau from TS20 to P7 with a high expression increase at P14. The Changepoint contrast again delivered a biased visualization. The change point might be recognized, but the overall trend was flawed. The Sequen contrast detected the change point as significant and above the relevance limit. The lower limit of the confidence interval exceeded the upper relevance limit with 2.15 [1.64; 2.66]. Finally, the McDermott contrast visualized the plateau in conjunction with the rise of expression with a point estimate of 2.01 [1.64; 2.37]. The last three confidence intervals were all above the relevance limit with an effect of 2.01, 2.93, and 2.63. There was no obvious expression change in the two biological examples, *Car9* expression in the developing liver and *Glut* expression in the developing kidney (Supplementary Section 2, Additional file 1). All three contrasts stayed within the relevance limits. The examples illustrated that both, biological visualisation and confidence intervals, are required to find biologically relevant change points.

### Simulation data

**Fig. 4:**
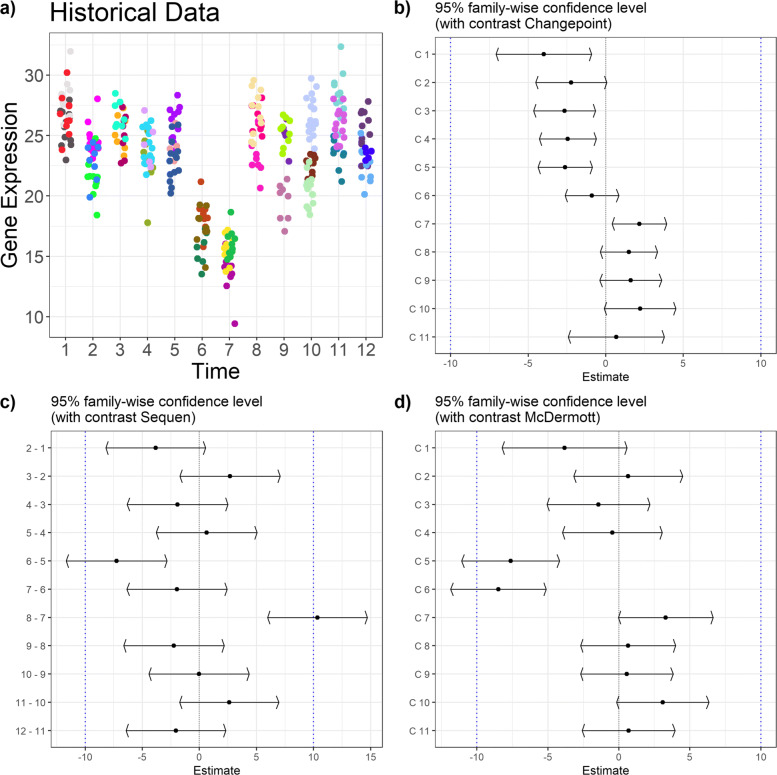
Confidence intervals of estimates from linear mixed model coupled with contrast matrix for historical data with a “partly dropped” change points. Figure **a**) shows the increasing points in time (x-axis) of the sampled historical data in association with gene expression (y-axis) with two expected change points. Each color is related to one mother mouse. Subfigures **b**), **c**) and **d**) show the estimates (x-axis) including confidence intervals for the observed contrasts (y-axis) with methods Changepoint, Sequen and McDermott, respectively. The blue line indicates the simulated effect

We simulated eleven simulation settings according to Fig. 1 and motivated by the biological examples. We fitted one linear mixed effect model on each of the simulated times series. These fitted models were then used for generalized linear hypothesis testing with three different contrast matrices. The results of interest were the mean difference and associated 95% confidence intervals. Depending on the contrast matrix used, the output suggested the presence or absence of change points (all simulation results in Supplementary Section 3, Additional file 1). We present here two out of the eleven simulated settings. Figures 3 and 4 show the course for the settings in Fig. 1c (“stepwise change”) and d (“partly dropped”). Tables 5 and 6 present respective the numeric values. We indicated the simulated change point by a gray row. The number of simulations was increased since we modelled expression decrease separately from expression increase.

**Table 5:**
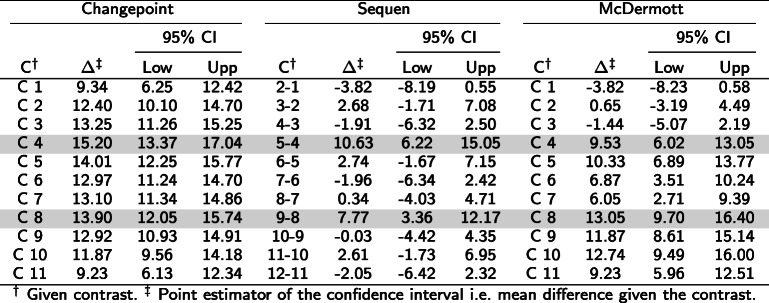
Contrasts and estimates to Fig. 3. The table shows the numeric values form the simulation for three change points. The C column indicates the contrast, the *Δ* the log mean change of the corresponding contrast C. The gray row indicates the predefined change point(s). A significant confidence interval does not include zero

^*†*^Given contrast. ^*‡*^ Point estimator of the confidence interval i.e. mean difference given the contrast

**Table 6:**
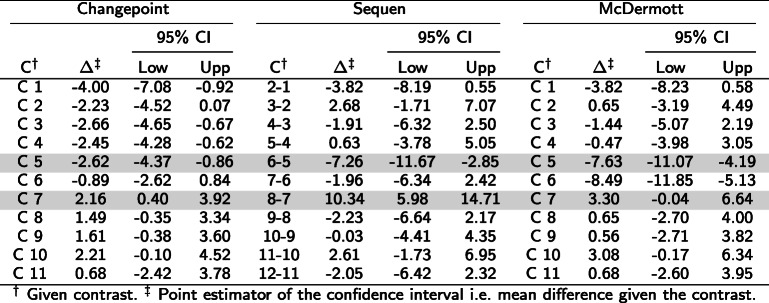
Contrasts and estimates to Fig. 4. The table shows the numeric values from the simulation for a “partly dropped” change point. The C column indicates the contrast, the *Δ* the log mean change of the corresponding contrast C. The gray row indicates the predefined change point(s). A significant confidence interval does not include zero

^*†*^Given contrast. ^*‡*^ Point estimator of the confidence interval i.e. mean difference given the contrast

Figure 3 showed a stepwise increase of expression (corresponding numeric values in Table 5) and we observed two distinct change points. For illustration purposes, we simulated the variance in such a way that a slight overlap of the observations occurred. The simulated effect was 10. Therefore, each rise/expression change increased the average expression by 10 (subplot a). In contrast to our assumption, the Changepoint contrast did not detect a change point (Fig. 3b, confidence intervals in Table 5). Hence, the name of the contrast was misleading - as was the position of all significant confidence intervals. The Sequen contrast delivered the change points correctly at contrasts 5-4 and 9-8. We were able to detect the change by the significant confidence intervals or visually by exceeding of the intervals. The direction of the change is also represented correctly. In addition, there is a slightly lower effect of 7.77 [3.36; 12.17] at the second compared to the first change point with 10.63 [6.22; 15.05] (subplot a). Hence, the Sequen contrast delivers the correct direction in conjunction with the correct effect estimates. Finally, the McDermott contrast mimicked the steps of the simulated data. Each rise at C4 and C8 could be observed by a stronger shift of the confidence intervals to the right with an effect of 9.53 [6.02; 13.05] and 13.05 [9.70; 16.40], respectively. Hence, position and the direction of the change point were both correct. The confidence interval itself was not on the same level because the single time points had slightly different means. These findings were also true for one and three positive change points (Supplementary Section 3 Fig. 6 and 8, Additional file 1) as well as one, two and three negative change points (Supplementary Section 3 Fig. 10, 11 and 12, Additional file 1). In summary, the Sequen and McDermott contrasts were able to detect the position and direction (Sequen) or the overall course (McDermott) of predefined change points.

Figure 4 presents a “partly dropped” change point (numeric values in Table 6). The expression was reduced at two time points before it is restored to the original values. In contrast to Fig. 3, the Changepoint contrast in Fig. 4 did deliver a change in the confidence interval plot but the indicated change of 2.16 [0.40; 3.92] at C7 did not mimic the simulated data. Again, the Changepoint contrast did not help to indicate the correct position or effect directions as it indicated a positive change instead of a negative one (decreased expression). The Sequen contrast indicated both change points at the correct position. The 6-5 and 8-7 contrasts were significant with an effect of -7.26 [-11.67; -2.85] and 10.34 [5.98; 14.71]. The direction was also correct. The first significant confidence interval had a negative effect, indicating the drop and the second significant confidence interval had a positive effect indicating the rise in expression. In comparison to the Sequen contrast, the McDermott contrast must be interpreted differently. Again, the two significant confidence intervals were indicating the area of change with two significant confidence intervals at C5 and C6 with an effect of -7.63 [-11.07; -4.19] and -8.49 [-11.85; -5.13]. However, the direction of the change must be calculated by the researcher. The McDermott contrast rather visualized the course than giving the concrete direction of the decrease/increase. Depending on the research question, Sequen or McDermott might be preferred. Supplementary Section 3 Fig. 13, Additional file 1, shows the extreme event of four time points with no expression and therefore no variance at those (numeric values of the confidence intervals in Supplementary Section 3 Table 13, Additional file 1). In this extreme scenario, all three contrasts delivered confidence intervals. Again, the Changepoint contrast pictured highly misleading directions and effects. We observed a lower plateau with a linear increase to another plateau, not at all emulating the course of the expression data at all. In contrast, the Sequen contrast correctly delivered the change point positions and directions at 5-4 and 9-8 with the effects of -8.76 [-12.62; -4.90] and 8.28 [4.43; 12.14]. The McDermott contrast had more biased confidence intervals. The drop was visualized by the contrast but the last confidence intervals falsely indicated a higher plateau of expression than at the beginning of the time course. In addition, the significant confidence intervals indicating the drop also falsely showed a steady decrease of the effect.

Finally, we simulated no change, linear increase, and linear decrease. Supplementary Section 3 Fig. 4, Additional file 1, shows the results of the “no change” simulation. None of the contrasts did detect any change points, presenting non-significant, overlapping confidence intervals. The results of “linear increase” and “decrease” are shown in Supplementary Section 3 Figs. 5 and 9, Additional file 1, respectively. The overall tendencies of the confidence intervals were the same in both settings. Supplementary Section 3 Fig. 5, Additional file 1, mirrored Supplementary Section Fig. 9, Additional file 1. The Changepoint contrast was significant for all confidence intervals with a strong effect. The point estimates were the same for nearly all confidence intervals. The Sequen contrast had some slightly significant confidence intervals. However, all confidence intervals overlapped, indicating no change in expression. The McDermott contrast mimicked the linear tendency of the expression data with its positive and negative trends. As all confidence intervals overlapped, we concluded that no change point was present.

A word of caution about the estimated effects and the direction of the effect. Our approach allows determining the point estimate of the difference between time points. Depending on the contrast, different effects will be reported. The preferred contrast is therefore highly dependent on the research question. While the Sequen contrast provides the point of change, the McDermott contrast visualizes the overall course of the change. In contrast, we cannot recommend the original Changepoint contrast for detection or assessment of the change point as its effect estimates are biased.

In summary, if Sequen or McDermott contrast matrices were applied and an actual change point was present in the simulated data, the confidence interval from the respective contrast was significant and no (or only a small) overlap with the confidence interval of the preceding contrast occurred. When there was no change point, the 95% confidence intervals for each contrast were either not significant or they overlapped with the confidence interval of the preceding contrast. The respective patterns can be observed in a more or less defined way on all simulated data for the Sequen and McDermott contrasts. The Changepoint contrast cannot be recommended for the detection of a change point in any simulation setting. Overall, we suggest using McDermott’s method to determine if there is a significant change within the time frame, while Sequen could be applied to determine the specific change point(s) and their direction(s).

## Discussion

In a classical longitudinal design, each patient is examined at each inter-dependent time point. In this study, we examined a different counterintuitive setting: The time points are independent as the intervention on the mice is lethal and the observations (gene expression in the litters’ organs) at each time point are correlated, resulting in a mixture of dependent and independent data structures at one time point. We solved the research question looking for change points in this experimental setting by using multiple contrast tests and by visualizing the change point with simultaneous confidence intervals. We investigated three contrasts which differ in the research questions they can answer: Should a single change point be found, or should the overall course rather be pictured? The Sequen contrast answers the first, the McDermott the second. The Changepoint contrast gives a clearly biased visualization and is unable to correctly determine change points in our setting. To summarize, we used generalized hypothesis testing with linear mixed effect models using various contrast matrices to detect change points in historical data of gene expression levels with independent and dependent data points.

A connected question is how long such a time line could be to still be able to detect differences. As generalized hypothesis testing was applied, it automatically adjusted locally for multiple testing. Therefore, for each model, the respective significance level was met. The number of time points minus one comparison was evaluated for all chosen contrasting methods. The higher the number of time points, the more contrasts were tested, leading to a stricter change point selection but also higher run times. In our method section, we only give an approximation of the theoretical maximal length of historical data because the main aim of our work was to identify the most informative contrast test for detecting a given change point pattern. Surprisingly, the Sequen and McDermott contrasts were found which both intuitively were not our first choice. In future work, the borders of the maximal number of time points and multiplicity adjustment approaches [28, 29] will be examined in more detail.

We have discussed the possible length of historical data in terms of significance. Thus, if a confidence interval is significant, we would assume a change point. However, in the biological example data, we could also define a relevance threshold ranging from (just barely) significant to biologically relevant in our decision making. The proper choice of estimands, i.e., effect estimators, is embedded in a more general discussion of reproducibility. To date, the discussion of estimands has focused on drug development and clinical trials. Akacha et al. (2017) [30] notes that certain choices in statistical analysis can partially or completely blur the scientific question. The interested reader might read Mallinckrodt et al. (2019) [31] for a detailed discussion of estimands, estimators, and sensitivity analyses in clinical trials.

Many multiple contrast tests are well described in the literature as well as the application in statistical inference [22]. The most common contrast might be the all-pairs contrast (also known as the Tukey contrast), or the many-to-one contrast (also known as the Dunnett contrast). Other types of contrasts are not so widespread and known. Interestingly, the so-called Changepoint contrast does not deliver any change point in the context of our experimental design. We do not criticize its general approach but for our data, it does not deliver the best interpretable change point(s) in the context of confidence intervals. The Sequen and McDermott contrasts are both able to detect change points while answering slightly different questions. Sequen visualizes the point and direction of change, while McDermott visualizes the course of the change. Of note, if the mean differences in sequential contrasts seem to be significant but switch between plus and minus, one should evaluate whether there are multiple change points or just high fluctuations in the measured values. Consequently, although change points were detected by these methods, one should still check for validity and relevance. Using generalized hypothesis testing may be a prefilter but the final decision should still be made by an expert of the respective field based on the context of the study.

If we used a simple linear model without taking the nested litter/mother effects into account, the linear model would cause some type of overdispersion. In addition, our model would not reflect our true data structure. The results would include a high amount of false positive (non-existing) change points. Especially, if we decided only based on significance. As a drawback, the lme package sometimes has convergence or model fitting problems with small sample sizes. In some cases, the lmer() function displays a “is singular” warning that the estimated variance-covariance matrix has some entries of zero. Therefore, the matrix does not have a full rank. In these cases, it is possible that some standard errors are underestimated and results should be considered with care.

We presented four in-vivo expression data sets of developmental stages in mice. We decided to present different biological courses to provide evidence for its practical application: Two of the data sets did not show any abrupt changes, one first showed a steady increase over three time points, stayed at that level for some time and then increased again. The fourth data set showed no changes apart from two time points with a drastic drop in expression. The respective R code can be found in the Supplementary Section 4, Additional file 1, as well on our GitHub repository. Therefore, the presented application should easily be replicated by the interested scientist. In our study, we presented a solution for historical data with a limited number of observed genes. If the number of genes went into the hundreds, a visual inspection would not be feasible any longer. Hence, the scientist would have to sort the potential change points by effect size in comparison to the respective relevance limits and only visually inspect the top relevance hits. A pattern recognition on confidence intervals is open to further research.

## Conclusion

In summary, we showed that multiple contrast tests can be used for change point detection in historical data. Our application is special in the sense that the individual time points are independent of each other. Nevertheless, there is a dependent data structure within the individual developmental stages. We showed that generalized hypothesis testing with linear mixed-effect models can be used to detect change points in clustered expression data. We delivered an approximation of the maximally usable time points in the historical data and allow the researcher to define relevance thresholds to guide decision making by the effect estimators. Our algorithm is easily applicable in R. We tested three different contrast matrices and found Sequen to be the best to detect a change point at a concrete time point in the course. Confidence intervals delivered a good visualization of the position of the change point as well as an interpretable estimator of the strength and direction of the change. To determine if there is an overall significant change within the time frame, we suggest using McDermott’s method as it is good at detecting changes throughout the historical data course. Both methods can also be used in sequence to verify results from historical data: First McDermott for a general overview and then Sequen for a selective examination of (parts of) the course.

## Supplementary Information

**Additional file 1** Supplementary Material.

## Data Availability

Online as Supplementary Section 4, Additional file 1, and code chunks and further information are also available from https://github.com/msieg08/clustered_data_changepoint_detection.

## Abbreviations

Actb: *β*-Actin
Car9: carbonic anhydrase 9
Changepoint: Multiple contrast name see Table 1 Glut1: glucose transporter
McDermott: Multiple contrast name see Table 3 Sequen: Multiple contrast name see Table 2

## References

[1] Page ES (1954). Continuous inspection schemes. Biometrika.

[2] Lorden G (1971). Procedures for reacting to a change in distribution. Ann Math Stat.

[3] Rao CV, Swarupchand U (2009). Multiple comparison procedures - a note and a bibliography. J Stat.

[4] Bretz F, Hothorn LA (2003). Statistical analysis of monotone or non-monotone dose–response data from in vitro toxicological assays. Altern Lab Anim.

[5] Bretz F, Hsu J, Pinheiro J, Liu Y (2008). Dose finding–a challenge in statistics. Biom J: J Math Meth Biosci.

[6] Salas-Huetos A, James ER, Aston KI, Jenkins TG, Carrell DT, Yeste M (2019). The expression of mirnas in human ovaries, oocytes, extracellular vesicles, and early embryos: a systematic review. Cells.

[7] Hu B, Zheng L, Long C, Song M, Li T, Yang L, Zuo Y (2019). Emexplorer: a database for exploring time activation of gene expression in mammalian embryos. Open Biol.

[8] Frye M, Harada BT, Behm M, He C (2018). Rna modifications modulate gene expression during development. Science.

[9] Hasler M, Hothorn LA (2008). Multiple contrast tests in the presence of heteroscedasticity. Biom J: J Math Meth Biosci.

[10] Schaarschmidt F, Vaas L (2009). Analysis of trials with complex treatment structure using multiple contrast tests. HortScience.

[11] Kruppa J, Hothorn L (2021). A comparison study on modeling of clustered and overdispersed count data for multiple comparisons. J Appl Stat.

[12] Hothorn LA (2006). Multiple comparisons and multiple contrasts in randomized dose-response trials—confidence interval oriented approaches. J Biopharm Stat.

[13] Wasserstein RL, Schirm AL, Lazar NA (2019). Moving to a world beyond “p< 0.05”. Am Stat.

[14] Akacha M, Bretz F, Ohlssen D, Rosenkranz G, Schmidli H (2017). Estimands and their role in clinical trials. Stat Biopharm Res.

[15] Ratitch B, Bell J, Mallinckrodt C, Bartlett JW, Goel N, Molenberghs G, O’Kelly M, Singh P, Lipkovich I (2020). Choosing estimands in clinical trials: Putting the ich e9 (r1) into practice. Ther Innov Regul Sci.

[16] Kirschner KM, Kelterborn S, Stehr H, Penzlin JLT, Jacobi CLJ, Endesfelder S, Sieg M, Kruppa J, Dame C, Sciesielski LK (2022). Adaptation of the oxygen sensing system during lung development. Oxidative Med Cell Longev.

[17] Sneddon LU, Halsey LG, Bury NR (2017). Considering aspects of the 3rs principles within experimental animal biology. J Exp Biol.

[18] Theiler K (2013). The House Mouse: Atlas of Embryonic Development.

[19] Lewis DI (2019). Animal experimentation: Implementation and application of the 3rs. Emerg Top Life Sci.

[20] Goldfeld K, Wujciak-Jens J (2020). simstudy: Illuminating research methods through data generation. J Open Source Softw.

[21] Schad DJ, Vasishth S, Hohenstein S, Kliegl R (2020). How to capitalize on a priori contrasts in linear (mixed) models: A tutorial. J Mem Lang.

[22] Bretz F, Hothorn T, Westfall P (2016). Multiple Comparisons Using R.

[23] Hothorn T, Bretz F, Westfall P (2008). Simultaneous inference in general parametric models. Biom J: J Math Meth Biosci.

[24] Bates D, Mächler M, Bolker B, Walker S. Fitting linear mixed-effects models using lme4. arXiv preprint arXiv:1406.5823. 2014.

[25] Mcdermott MP (1999). Generalized orthogonal contrast tests for homogeneity of ordered means. Can J Stat.

[26] Matuschek H, Kliegl R, Vasishth S, Baayen H, Bates D (2017). Balancing type i error and power in linear mixed models. J Mem Lang.

[27] Barr DJ, Levy R, Scheepers C, Tily HJ (2013). Random effects structure for confirmatory hypothesis testing: Keep it maximal. J Mem Lang.

[28] Alosh M, Bretz F, Huque M (2014). Advanced multiplicity adjustment methods in clinical trials. Stat Med.

[29] Li G, Taljaard M, Van den Heuvel ER, Levine MA, Cook DJ, Wells GA, Devereaux PJ, Thabane L (2017). An introduction to multiplicity issues in clinical trials: the what, why, when and how. Int J Epidemiol.

[30] Akacha M, Bretz F, Ruberg S (2017). Estimands in clinical trials–broadening the perspective. Stat Med.

[31] Mallinckrodt C, Molenberghs G, Lipkovich I, Ratitch B (2019). Estimands, Estimators and Sensitivity Analysis in Clinical Trials.

