# Supplementary material for: Change point detection for clustered expression data
Source: BMC Genomics. 2022 Jul 6;23:491. doi: 10.1186/s12864-022-08680-9 (PMC9261071; doi:10.1186/s12864-022-08680-9)
Supplement: Supplementary file 1 — Additional file 1 Supplementary Material. [file 12864_2022_8680_MOESM1_ESM.pdf]

Additional File 1 - Supplementary Material

Change point detection for clustered expression data

Miriam Sieg[1*], Lina Katrin Sciesielski[2], Karin Michaela Kirschner[3], and Jochen Kruppa[4]

[1]Institute of Biometry and Clinical Epidemiology, Charité - Universitätsmedizin Berlin
[2]Department of Neonatology, Charité - Universitätsmedizin Berlin
[3]Institute of Translational Physiology, Charité - Universitätsmedizin Berlin
[4]Hochschule Osnabrück - University of Applied Sciences
[*]corresponding author

Contents

1 Overview of the Supplementary Material **3**

2 Biological Expression Example Data **4**

2.1 Additional Information on the Biological Expression Example Data . . . 4

2.2 Additional Figures . . . 5

2.3 Additional Tables . . . 8

3 Simulation Setting **10**

3.1 Additional Figures . . . 10

3.2 Additional Tables . . . 21

4 Analysis in R **27**

5 Effect of the Litter Variance **31**

6 Flowchart of the Method **33**

1 Overview of the Supplementary Material

In the following additional material of the paper is provided. We show in additional figures to the biological data and additional figures to the simulation settings. In addition, we present also some R code.

Supplementary Section 2 provides three additional biological data sets.

Supplementary Section 3 provides additional information on the simulation setting. We repeated all settings to have a better overview over all simulations. Therefore, some figures are duplicated in the paper and in the supplement.

Supplementary Section 4 presents additional R code for our analysis. Please also consider the GitHub repository for the direct access to the R code: https://github.com/msieg08/clustered_data_changepoint_detection

Supplementary Section 5 presents a small simulation study with different different litter variance on the course of the confidence intervals

Supplementary Section 6 shows a flowchart of the methods from the simulation to the final figures.

2 Biological Expression Example Data

2.1 Additional Information on the Biological Expression Example Data

The data structure was the same for all four data sets. Each data set consisted of gene expression data from multiple samples determined at 12 fixed time points plus the adult stage. The time points represent different developmental stages. The gene expression information was constrained to one gene measured in one organ per time series. Expression of a specific gene in a specific organ was measured in multiple mouse pups by multiple mothers from twelve days after coitus (E12, Theiler Stage TS20) onwards. The 12 fixed time points contained two embryonic, four fetal, six postnatal and the adult stage(s). No pup was included twice and each mother only had one litter, i.e. at each time point, the litters originated from different mothers. The variance introduced from a varying litter is called the litter effect. Not including this information in the final model could lead to overdispersion [1]. From a statistical point of view, this means expression data gained from the same litter was dependent, but was independent between the litters. Hence, at each time point the data consisted of both dependent and independent data points. Additionally, expression information between different time points was independent.

2.2 Additional Figures

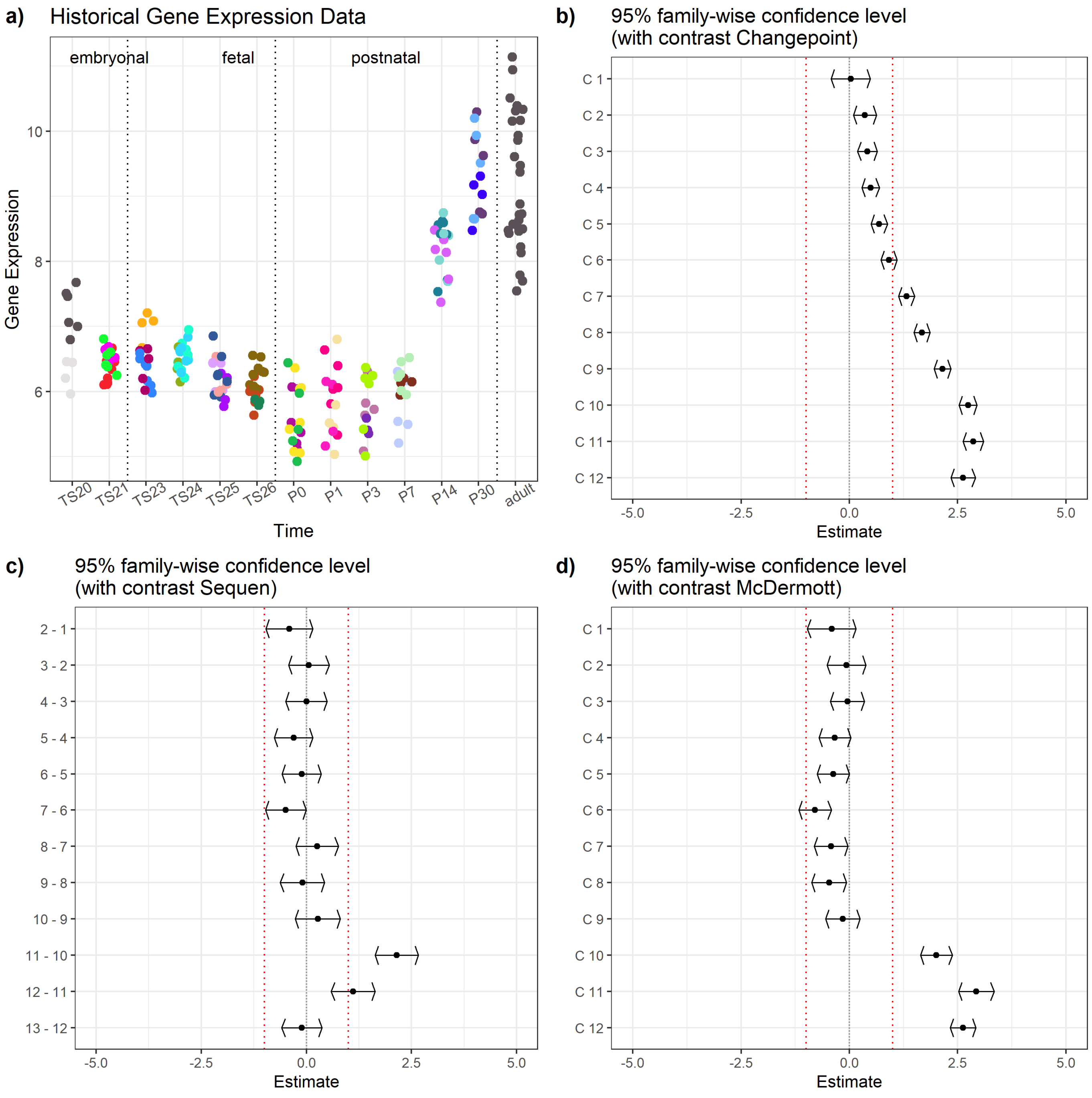

Supplementary Figure 1: **Biological example data for *Car9* expression in the developing kidney.** Subplot a) shows the biological example data set. Each point in time on the x-axis represents a development stage. The development stages are independent. Each point represents a pup and each color a mother animal. The pups are nested into the mothers. We added three broader development stages: embryonal, fetal, and postnatal. Subplot b) shows the result of the Changepoint contrast, subplot c) the confidence intervals of the Sequen contrast, and subplot c) the confidence intervals of the McDermott contrast. The red scattered line indicates the chosen limits of biological relevance. The model fit shows converting problems.

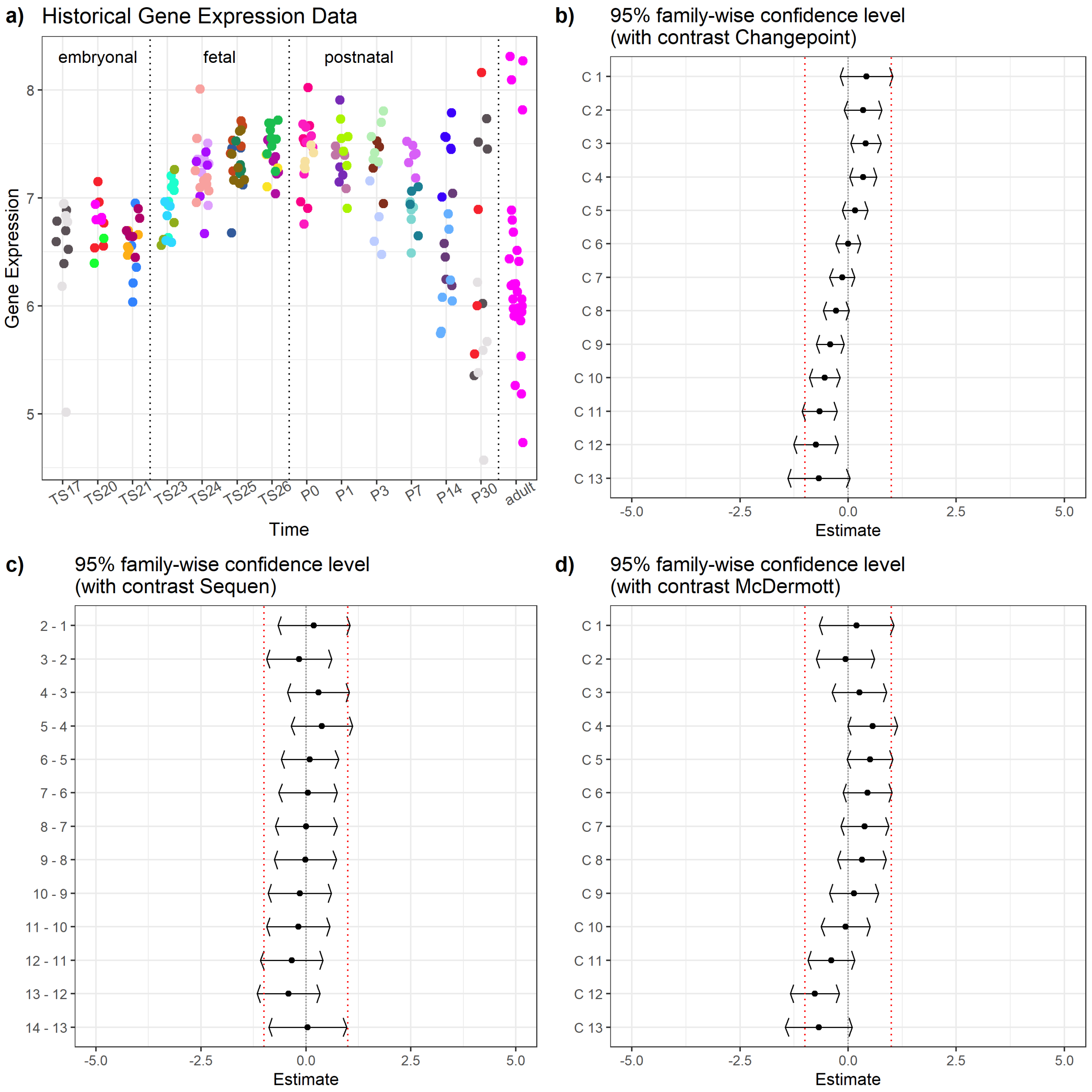

Supplementary Figure 2: **Biological example data for *Car9* expression in the developing liver.** Subplot a) shows the biological example data set. Each point in time on the x-axis represents a development stage. The development stages are independent. Each point represents a pup and each color a mother animal. The pups are nested into the mothers. We added three broader development stages: embryonal, fetal, and postnatal. Subplot b) shows the result of the Changepoint contrast, subplot c) the confidence intervals of the Sequen contrast, and subplot c) the confidence intervals of the McDermott contrast. The red scattered line indicates the chosen limits of biological relevance.

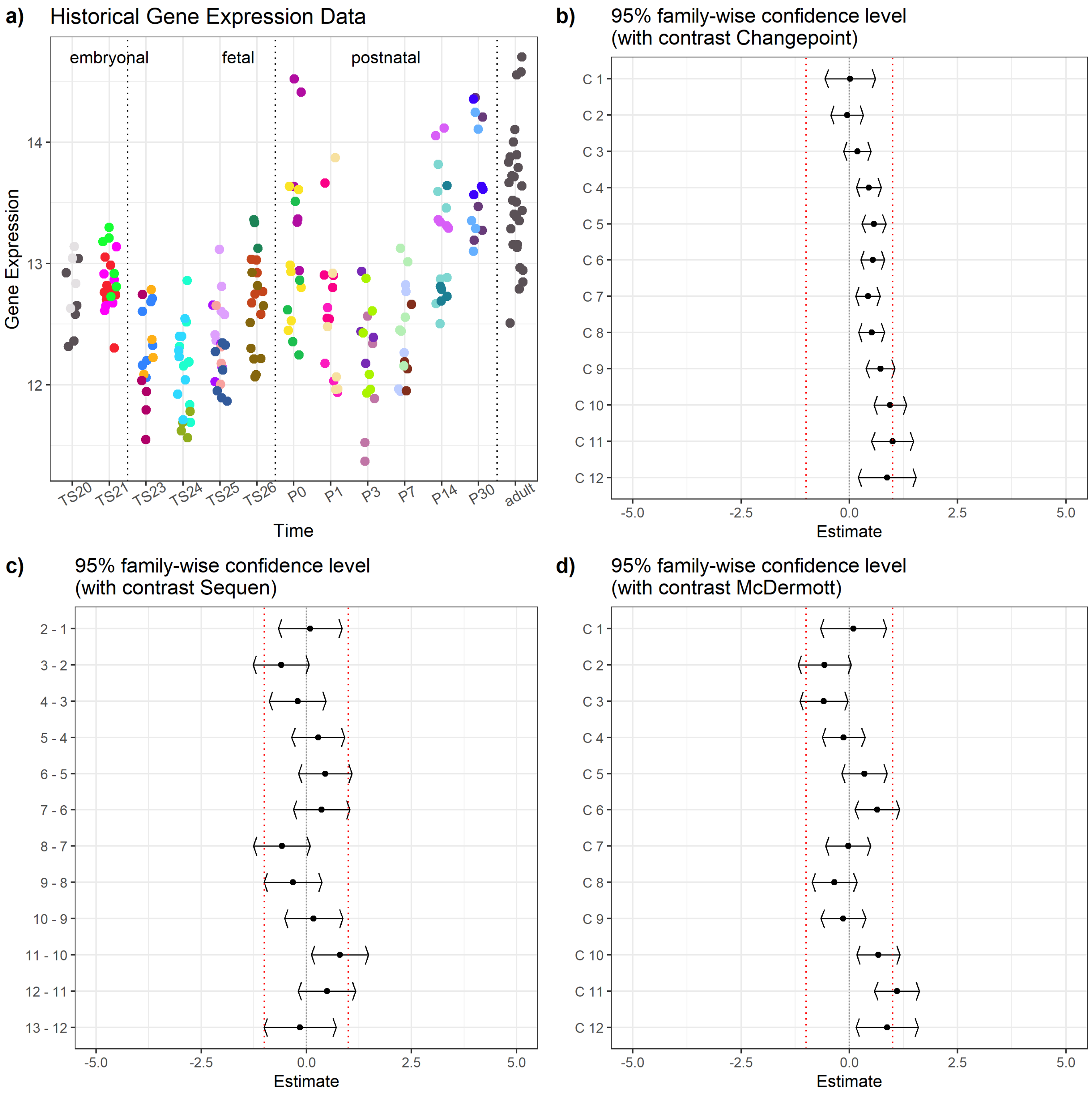

Supplementary Figure 3: **Biological example data for *Glut1* expression in the developing kidney.** Subplot a) shows the biological example data set (log-transformed). Each point in time on the x-axis represents a development stage. Each data point represents a pup and each color a mother animal. The pups are nested into the mothers. We added three broader development stages (embryonal, fetal, postnatal) for easier reference. The subplots show the confidence intervals of the Changepoint contrast (b), Sequen contrast (c), and McDermott contrast (d). The red scattered line indicates the chosen limits of biological relevance.

2.3 Additional Tables

Supplementary Table 1: Contrasts and estimates of figure 1. The table shows the numeric values form the kidney car9 example. The C column indicates the contrast, the Δ the log mean change of the corresponding contrast C. The gray row indicates a possible change point by visual inspection of figure 1. A significant confidence interval does not include zero.

Changepoint				Sequen				McDermott			
		95% CI				95% CI				95% CI	
C†	Δ‡	Low	Upp	C†	Δ‡	Low	Upp	C†	Δ‡	Low	Upp
C 1	0.03	-0.42	0.48	2 - 1	-0.41	-0.97	0.15	C 1	-0.41	-0.97	0.16
C 2	0.36	0.09	0.62	3 - 2	0.06	-0.42	0.54	C 2	-0.07	-0.52	0.38
C 3	0.42	0.19	0.64	4 - 3	-0.00	-0.49	0.49	C 3	-0.05	-0.44	0.35
C 4	0.49	0.29	0.69	5 - 4	-0.30	-0.76	0.15	C 4	-0.34	-0.70	0.03
C 5	0.69	0.50	0.88	6 - 5	-0.11	-0.58	0.35	C 5	-0.37	-0.74	0.00
C 6	0.91	0.73	1.10	7 - 6	-0.49	-0.97	-0.01	C 6	-0.79	-1.17	-0.42
C 7	1.32	1.13	1.50	8 - 7	0.26	-0.25	0.76	C 7	-0.42	-0.82	-0.03
C 8	1.68	1.49	1.86	9 - 8	-0.09	-0.62	0.43	C 8	-0.47	-0.87	-0.07
C 9	2.15	1.95	2.35	10 - 9	0.27	-0.26	0.81	C 9	-0.15	-0.55	0.25
C 10	2.74	2.53	2.95	11 - 10	2.15	1.64	2.66	C 10	2.01	1.64	2.37
C 11	2.86	2.62	3.10	12 - 11	1.11	0.59	1.64	C 11	2.93	2.52	3.34
C 12	2.63	2.34	2.91	13 - 12	-0.11	-0.59	0.37	C 12	2.63	2.33	2.92

† Given contrast.

‡ Point estimator of the confidence interval i.e. mean difference given the contrast.

Supplementary Table 2: Contrasts and estimates of figure 2. The table shows the numeric values from the liver car9 example. The C column indicates the contrast, the Δ the log mean change of the corresponding contrast C. A significant confidence interval does not include zero.

Changepoint				Sequen				McDermott			
		95% CI				95% CI				95% CI	
C†	Δ‡	Low	Upp	C†	Δ‡	Low	Upp	C†	Δ‡	Low	Upp
C 1	0.42	-0.19	1.04	2 - 1	0.19	-0.67	1.05	C 1	0.19	-0.67	1.06
C 2	0.34	-0.09	0.78	3 - 2	-0.16	-0.94	0.61	C 2	-0.06	-0.74	0.61
C 3	0.41	0.06	0.76	4 - 3	0.30	-0.44	1.04	C 3	0.26	-0.37	0.89
C 4	0.35	0.03	0.66	5 - 4	0.38	-0.35	1.11	C 4	0.57	-0.01	1.15
C 5	0.16	-0.14	0.46	6 - 5	0.09	-0.59	0.78	C 5	0.50	-0.02	1.03
C 6	0.00	-0.29	0.29	7 - 6	0.05	-0.65	0.75	C 6	0.45	-0.12	1.02
C 7	-0.14	-0.43	0.15	8 - 7	0.01	-0.73	0.74	C 7	0.38	-0.17	0.94
C 8	-0.28	-0.58	0.03	9 - 8	-0.01	-0.75	0.73	C 8	0.32	-0.25	0.88
C 9	-0.42	-0.74	-0.10	10 - 9	-0.15	-0.90	0.61	C 9	0.14	-0.43	0.70
C 10	-0.54	-0.90	-0.19	11 - 10	-0.18	-0.94	0.58	C 10	-0.06	-0.62	0.51
C 11	-0.66	-1.07	-0.26	12 - 11	-0.34	-1.08	0.41	C 11	-0.39	-0.93	0.15
C 12	-0.74	-1.26	-0.23	13 - 12	-0.41	-1.16	0.33	C 12	-0.77	-1.34	-0.20
C 13	-0.68	-1.40	0.04	14 - 13	0.04	-0.89	0.97	C 13	-0.68	-1.45	0.09

† Given contrast.

‡ Point estimator of the confidence interval i.e. mean difference given the contrast.

Supplementary Table 3: Contrasts and estimates of figure 3. The table shows the numeric values from the *Glut1* example data from liver. The C column indicates the contrast, the Δ the log mean change of the corresponding contrast C. The gray row indicates a possible change point by visual inspection of figure 3. A significant confidence interval does not include zero.

Changepoint				Sequen				McDermott			
		95% CI				95% CI				95% CI	
C†	Δ‡	Low	Upp	C†	Δ‡	Low	Upp	C†	Δ‡	Low	Upp
C 1	0.02	-0.56	0.60	2 - 1	0.09	-0.66	0.85	C 1	0.09	-0.67	0.85
C 2	-0.05	-0.43	0.32	3 - 2	-0.60	-1.27	0.07	C 2	-0.57	-1.19	0.04
C 3	0.18	-0.14	0.50	4 - 3	-0.21	-0.88	0.47	C 3	-0.59	-1.15	-0.03
C 4	0.45	0.16	0.74	5 - 4	0.28	-0.35	0.91	C 4	-0.13	-0.63	0.36
C 5	0.57	0.29	0.84	6 - 5	0.45	-0.19	1.09	C 5	0.35	-0.18	0.88
C 6	0.54	0.26	0.82	7 - 6	0.36	-0.31	1.03	C 6	0.65	0.13	1.16
C 7	0.43	0.14	0.71	8 - 7	-0.58	-1.26	0.09	C 7	-0.03	-0.55	0.49
C 8	0.51	0.21	0.82	9 - 8	-0.32	-1.01	0.37	C 8	-0.35	-0.87	0.18
C 9	0.72	0.38	1.05	10 - 9	0.17	-0.52	0.86	C 9	-0.14	-0.66	0.38
C 10	0.94	0.56	1.32	11 - 10	0.80	0.12	1.48	C 10	0.67	0.17	1.17
C 11	1.00	0.51	1.48	12 - 11	0.49	-0.19	1.17	C 11	1.10	0.58	1.62
C 12	0.87	0.20	1.54	13 - 12	-0.15	-1.01	0.71	C 12	0.87	0.15	1.59

† Given contrast.

‡ Point estimator of the confidence interval i.e. mean difference given the contrast.

3 Simulation Setting

3.1 Additional Figures

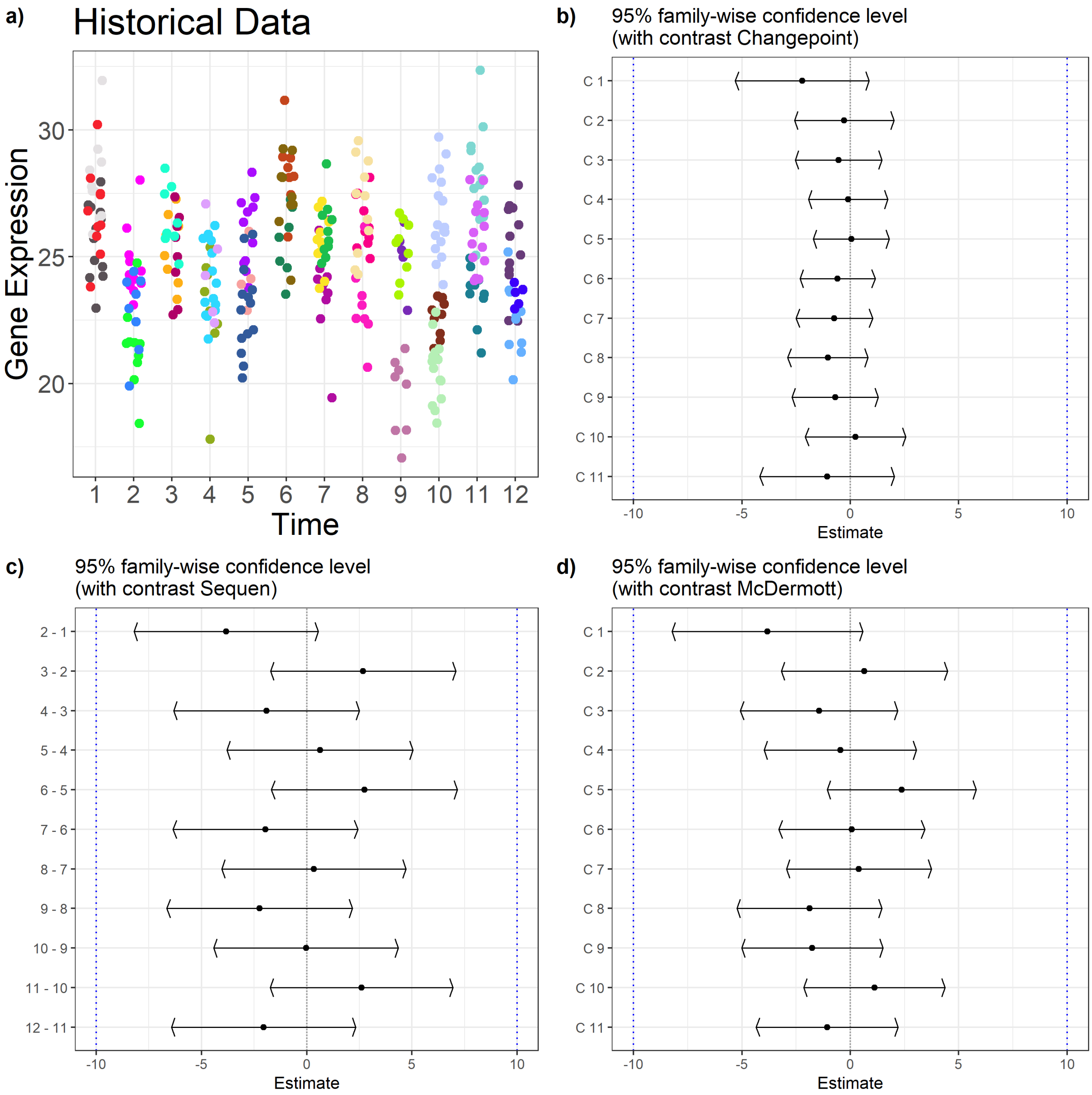

Supplementary Figure 4: **Confidence intervals of estimates from linear mixed model coupled with contrast matrix for historical data with no change point.** Figure a) shows the increasing points in time (x-axis) of the sampled historical data in association with gene expression activity (y-axis) with no expected change point. Each color is related to one mother mouse. Subfigures b), c) and d) show the estimates (x-axis) including confidence intervals for the observed contrasts (y-axis) with methods Changepoint, Sequen and McDermott, respectively.

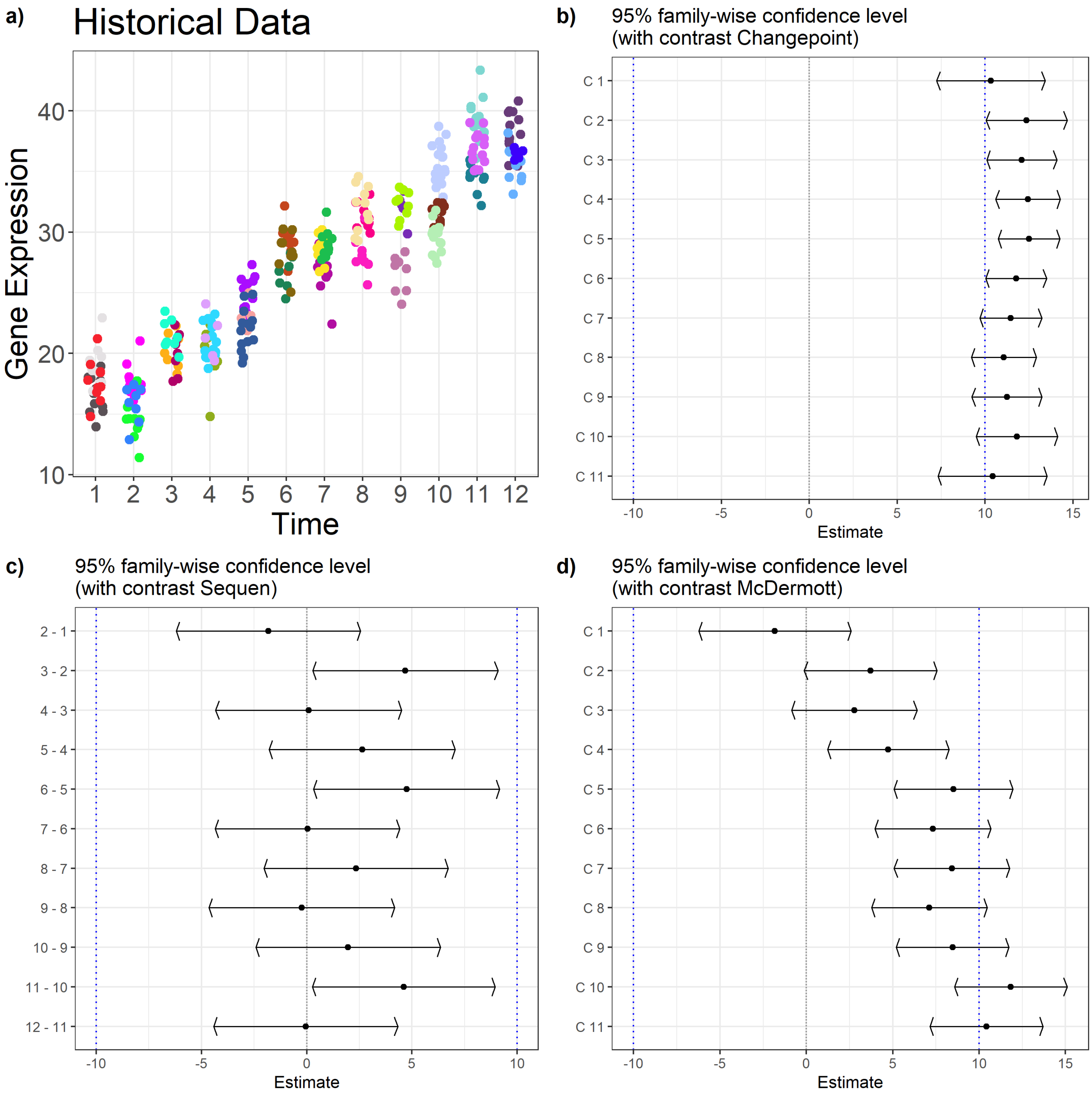

Supplementary Figure 5: **Confidence intervals of estimates from linear mixed model coupled with contrast matrix for historical data with a positive linear trend.** Figure a) shows the increasing points in time (x-axis) of the sampled historical data in association with gene expression activity (y-axis) with no expected change point. Each color is related to one mother mouse. Subfigures b), c) and d) show the estimates (x-axis) including confidence intervals for the observed contrasts (y-axis) with methods Changepoint, Sequen and McDermott, respectively.

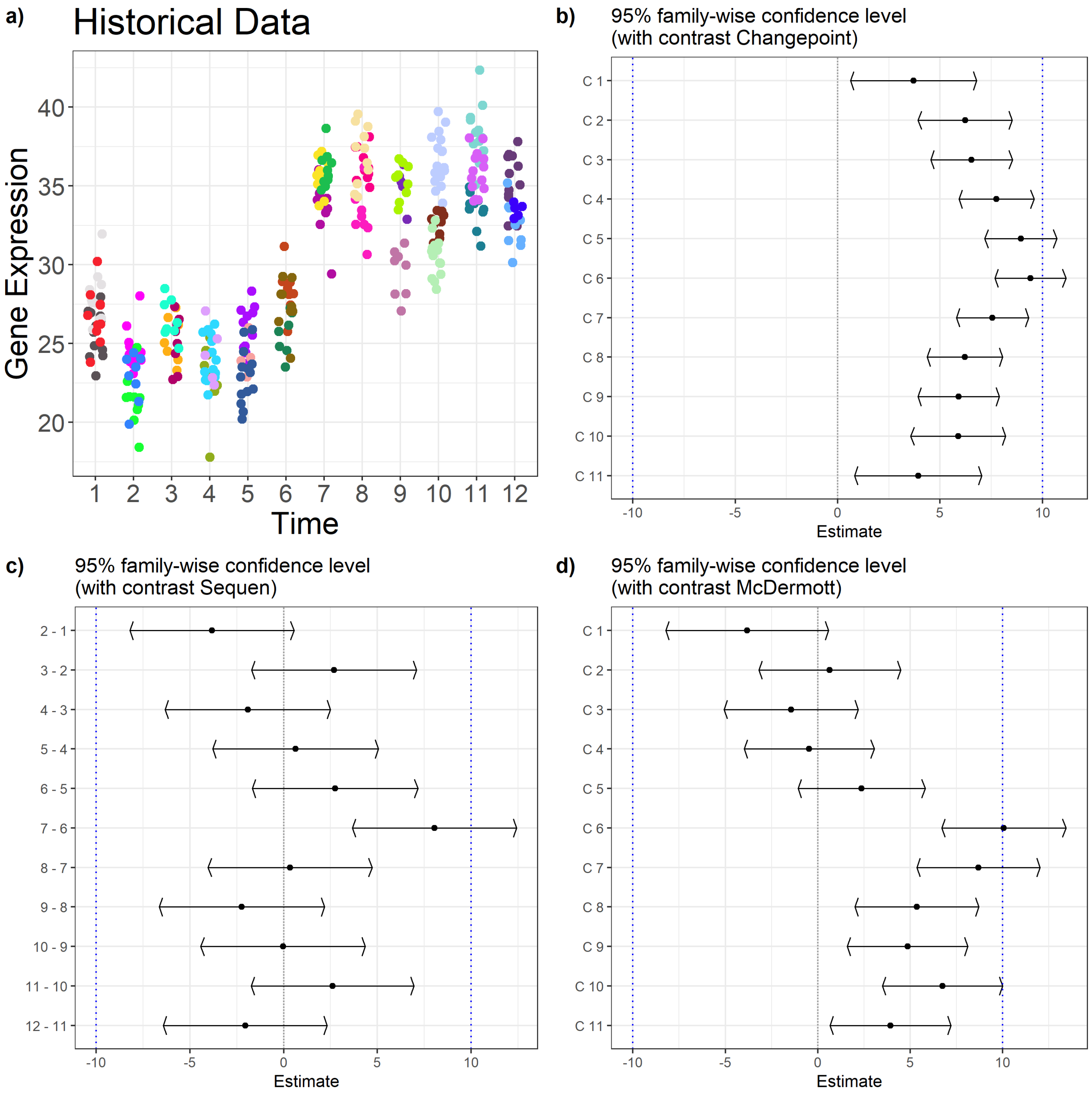

Supplementary Figure 6: **Confidence intervals of estimates from linear mixed model coupled with contrast matrix for historical data with one positive change point.** Figure a) shows the increasing points in time (x-axis) of the sampled historical data in association with gene expression activity (y-axis) with no expected change point. Each color is related to one mother mouse. Subfigures b), c) and d) show the estimates (x-axis) including confidence intervals for the observed contrasts (y-axis) with methods Changepoint, Sequen and McDermott, respectively.

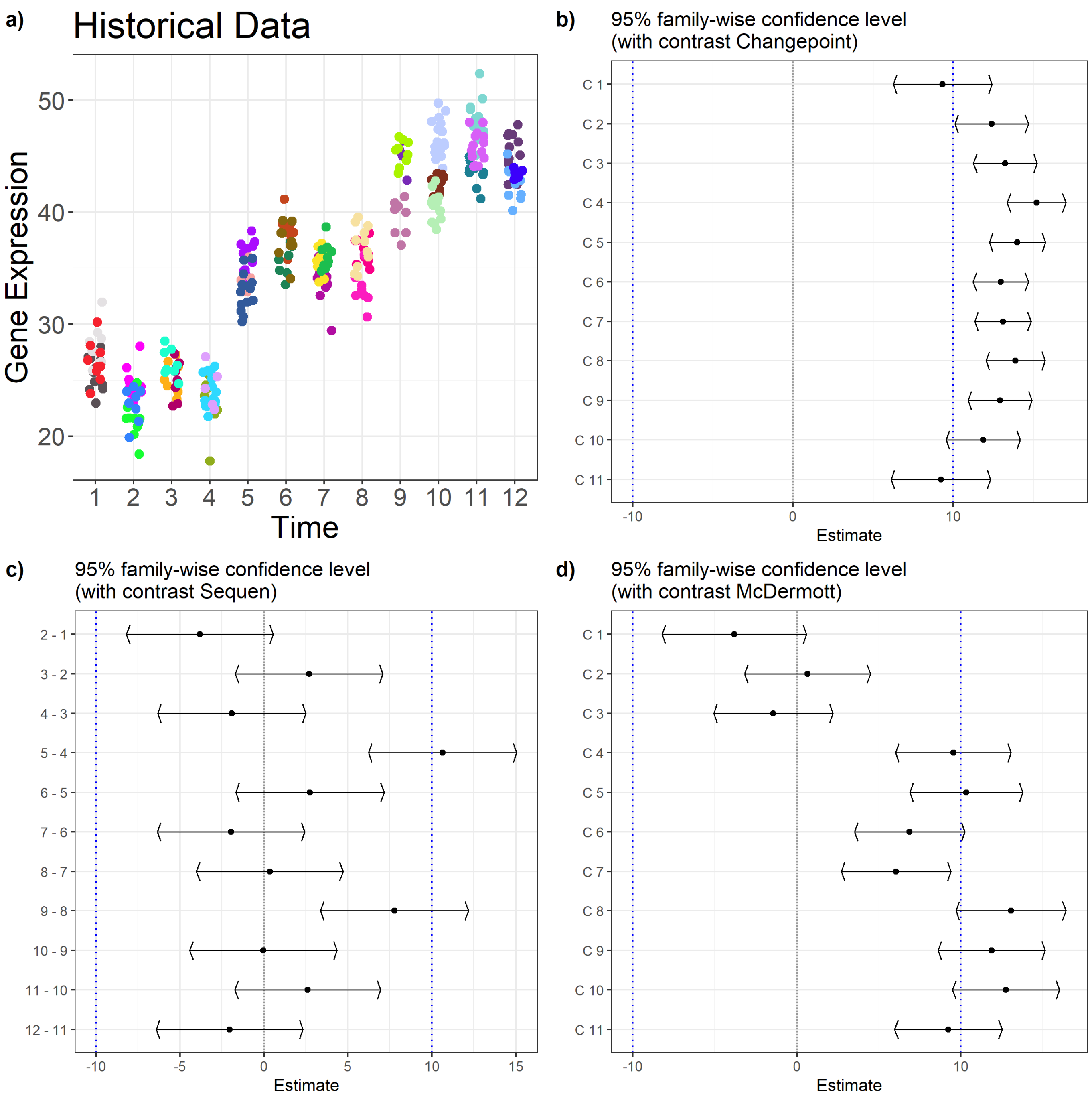

Supplementary Figure 7: **Confidence intervals of estimates from linear mixed model coupled with contrast matrix for historical data with two positive change points.** Figure a) shows the increasing points in time (x-axis) of the sampled historical data in association with gene expression activity (y-axis) with no expected change point. Each color is related to one mother mouse. Subfigures b), c) and d) show the estimates (x-axis) including confidence intervals for the observed contrasts (y-axis) with methods Changepoint, Sequen and McDermott, respectively.

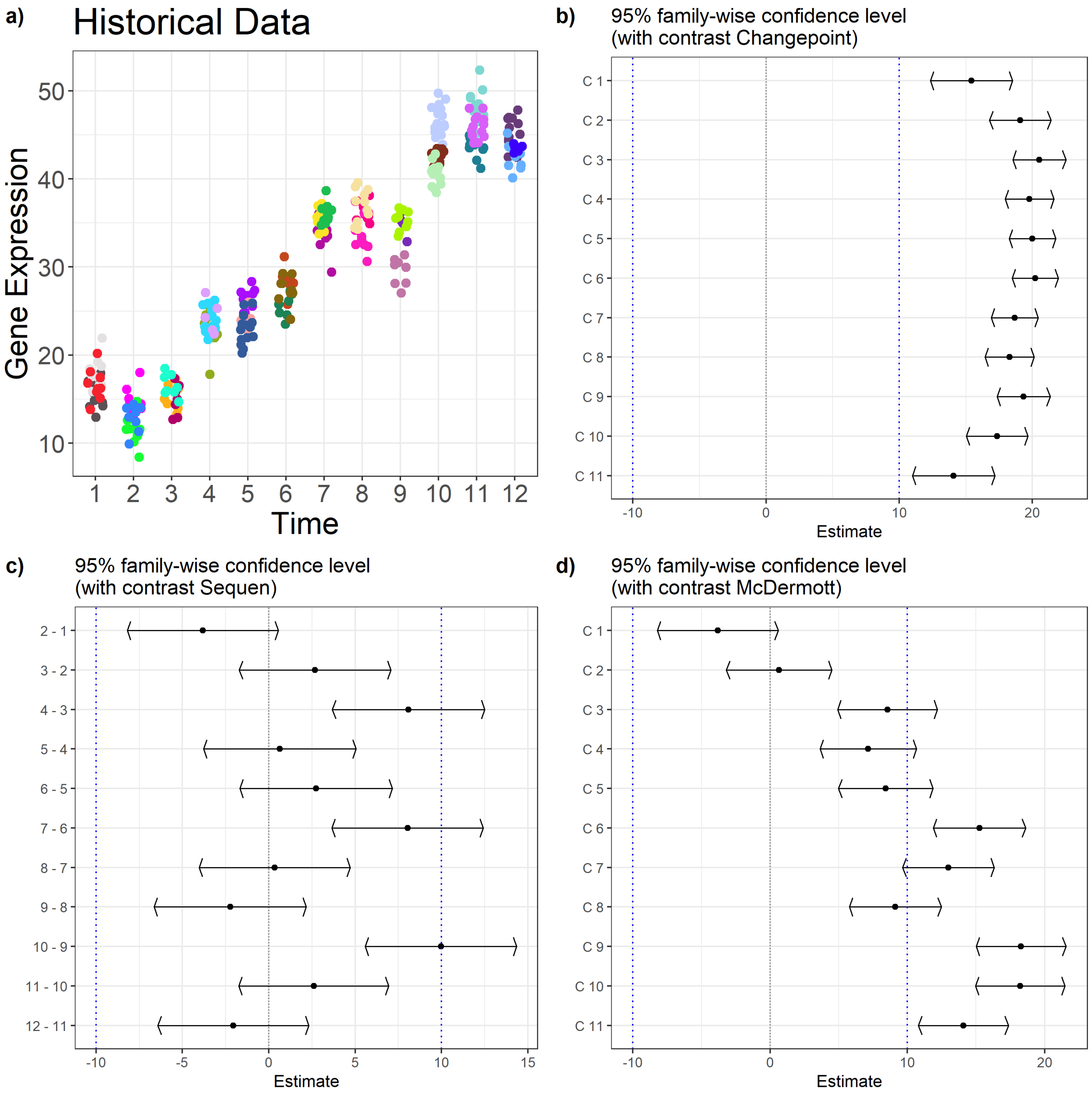

Supplementary Figure 8: **Confidence intervals of estimates from linear mixed model coupled with contrast matrix for historical data with three positive change points.** Figure a) shows the increasing points in time (x-axis) of the sampled historical data in association with gene expression activity (y-axis) with no expected change point. Each color is related to one mother mouse. Subfigures b), c) and d) show the estimates (x-axis) including confidence intervals for the observed contrasts (y-axis) with methods Changepoint, Sequen and McDermott, respectively.

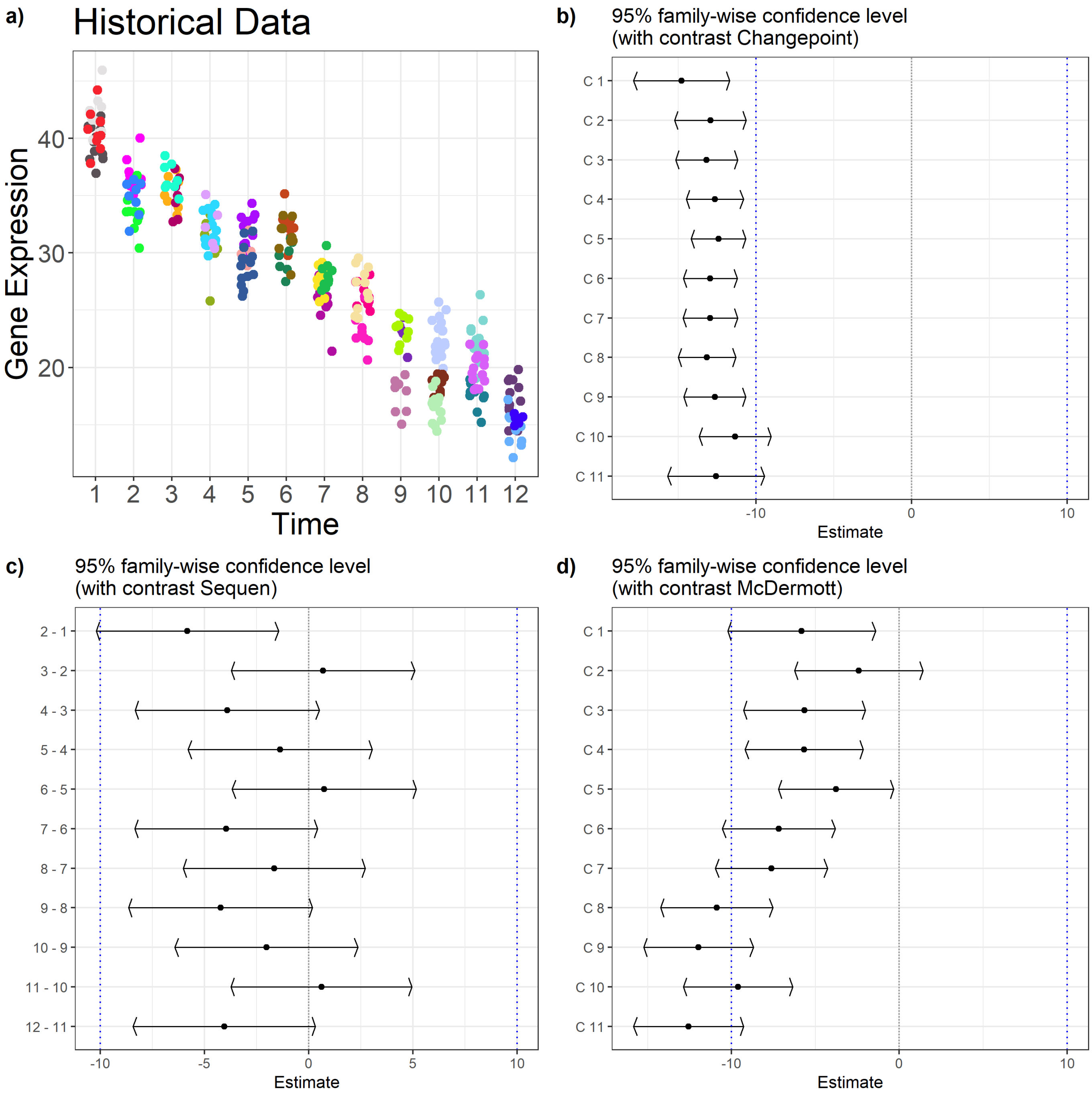

Supplementary Figure 9: **Confidence intervals of estimates from linear mixed model coupled with contrast matrix for historical data with a negative linear trend.** Figure a) shows the increasing points in time (x-axis) of the sampled historical data in association with gene expression activity (y-axis) with no expected change point. Each color is related to one mother mouse. Subfigures b), c) and d) show the estimates (x-axis) including confidence intervals for the observed contrasts (y-axis) with methods Changepoint, Sequen and McDermott, respectively.

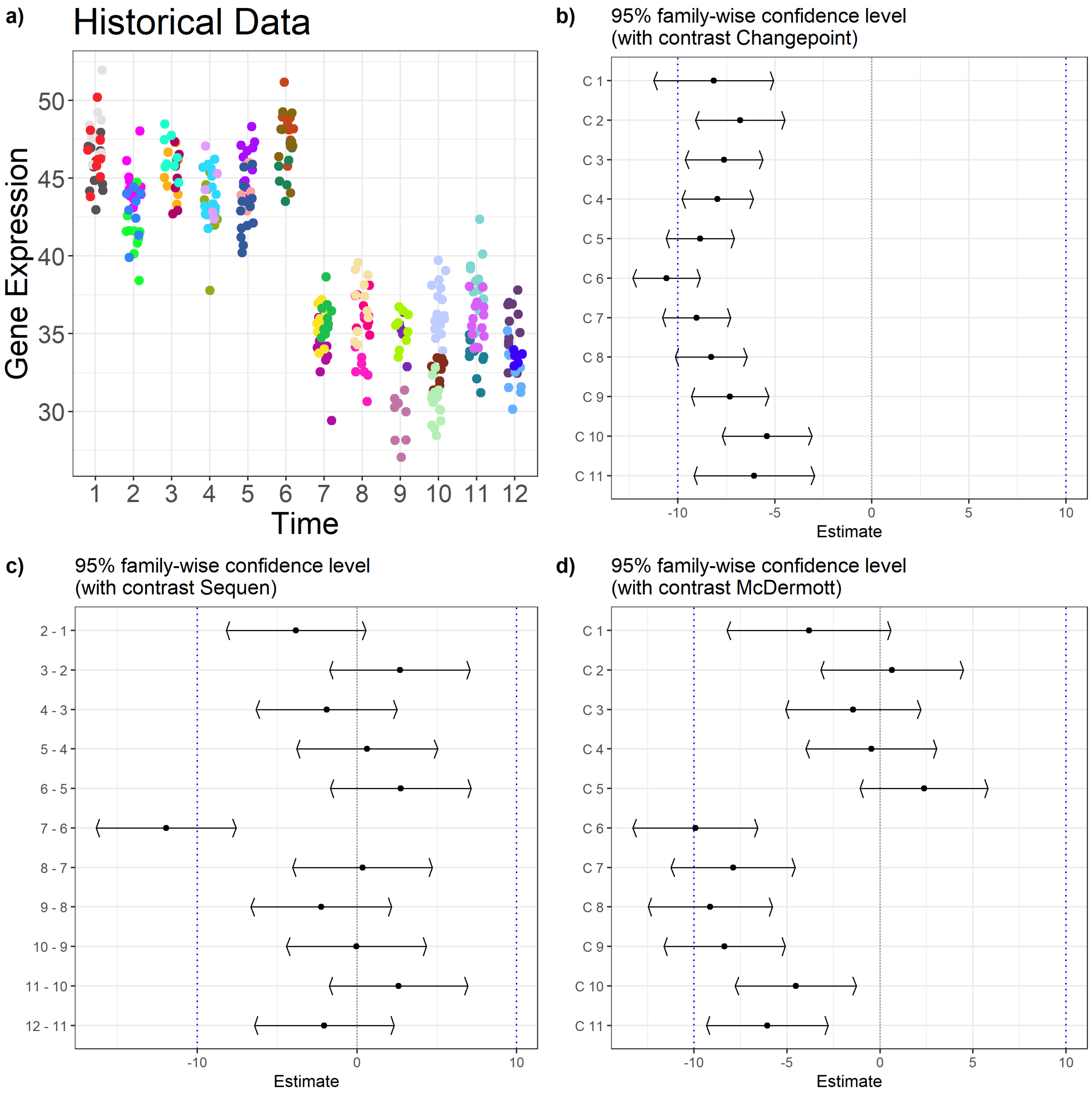

Supplementary Figure 10: **Confidence intervals of estimates from linear mixed model coupled with contrast matrix for historical data with one negative change point.** Figure a) shows the increasing points in time (x-axis) of the sampled historical data in association with gene expression activity (y-axis) with no expected change point. Each color is related to one mother mouse. Subfigures b), c) and d) show the estimates (x-axis) including confidence intervals for the observed contrasts (y-axis) with methods Changepoint, Sequen and McDermott, respectively.

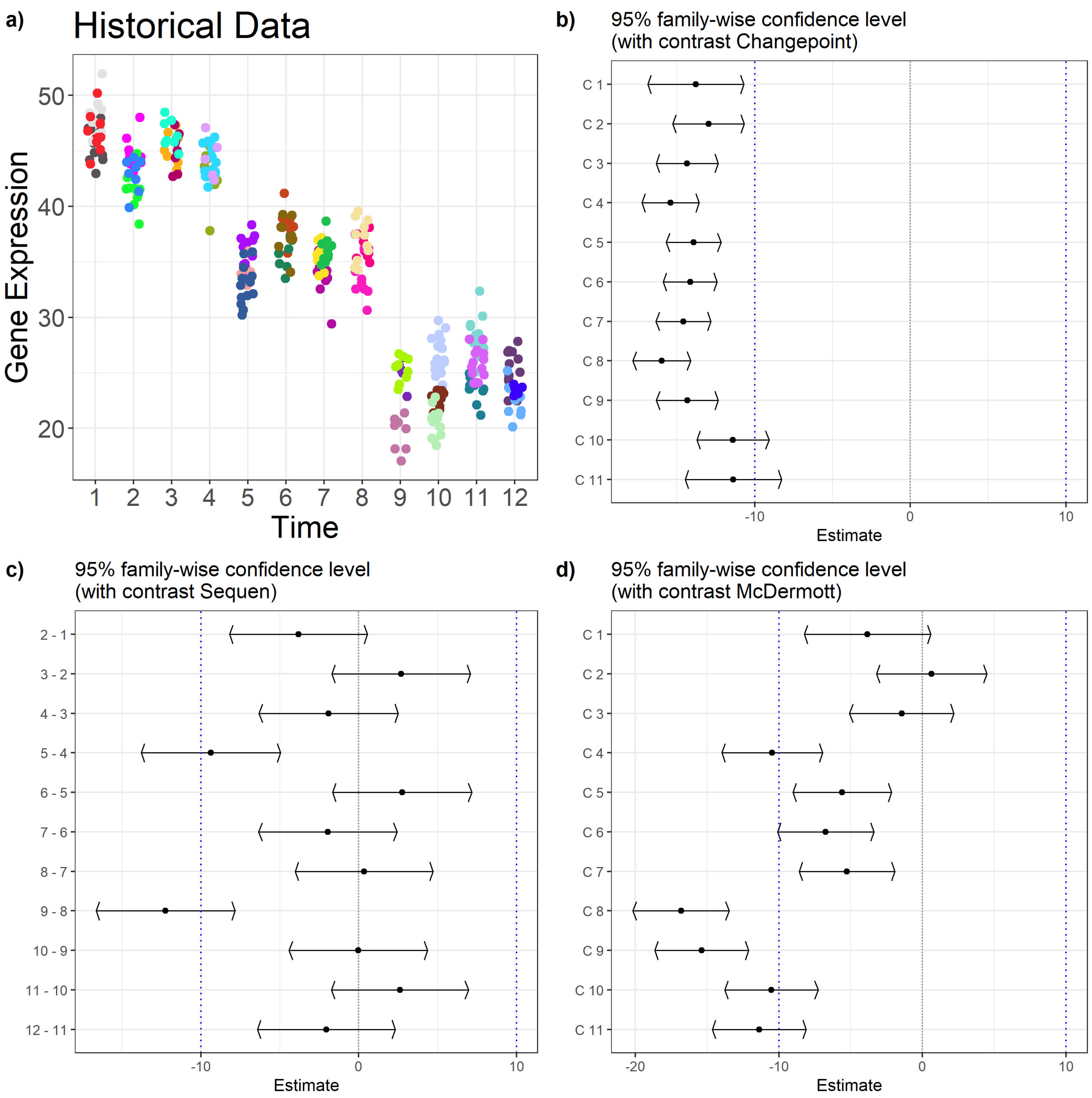

Supplementary Figure 11: **Confidence intervals of estimates from linear mixed model coupled with contrast matrix for historical data with two negative change point.** Figure a) shows the increasing points in time (x-axis) of the sampled historical data in association with gene expression activity (y-axis) with no expected change point. Each color is related to one mother mouse. Subfigures b), c) and d) show the estimates (x-axis) including confidence intervals for the observed contrasts (y-axis) with methods Changepoint, Sequen and McDermott, respectively.

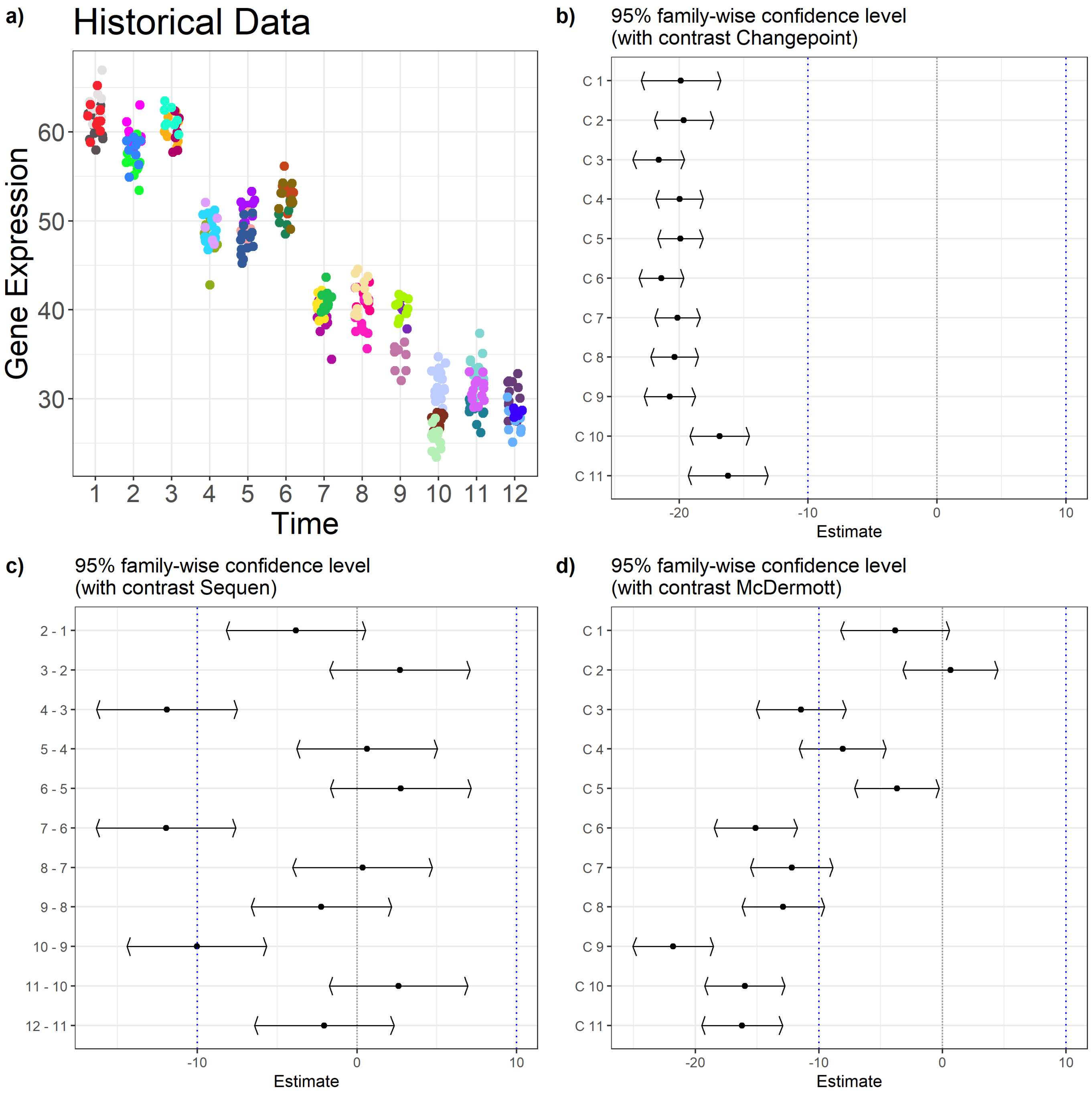

Supplementary Figure 12: **Confidence intervals of estimates from linear mixed model coupled with contrast matrix for historical data with three negative change point.** Figure a) shows the increasing points in time (x-axis) of the sampled historical data in association with gene expression activity (y-axis) with no expected change point. Each color is related to one mother mouse. Subfigures b), c) and d) show the estimates (x-axis) including confidence intervals for the observed contrasts (y-axis) with methods Changepoint, Sequen and McDermott, respectively.

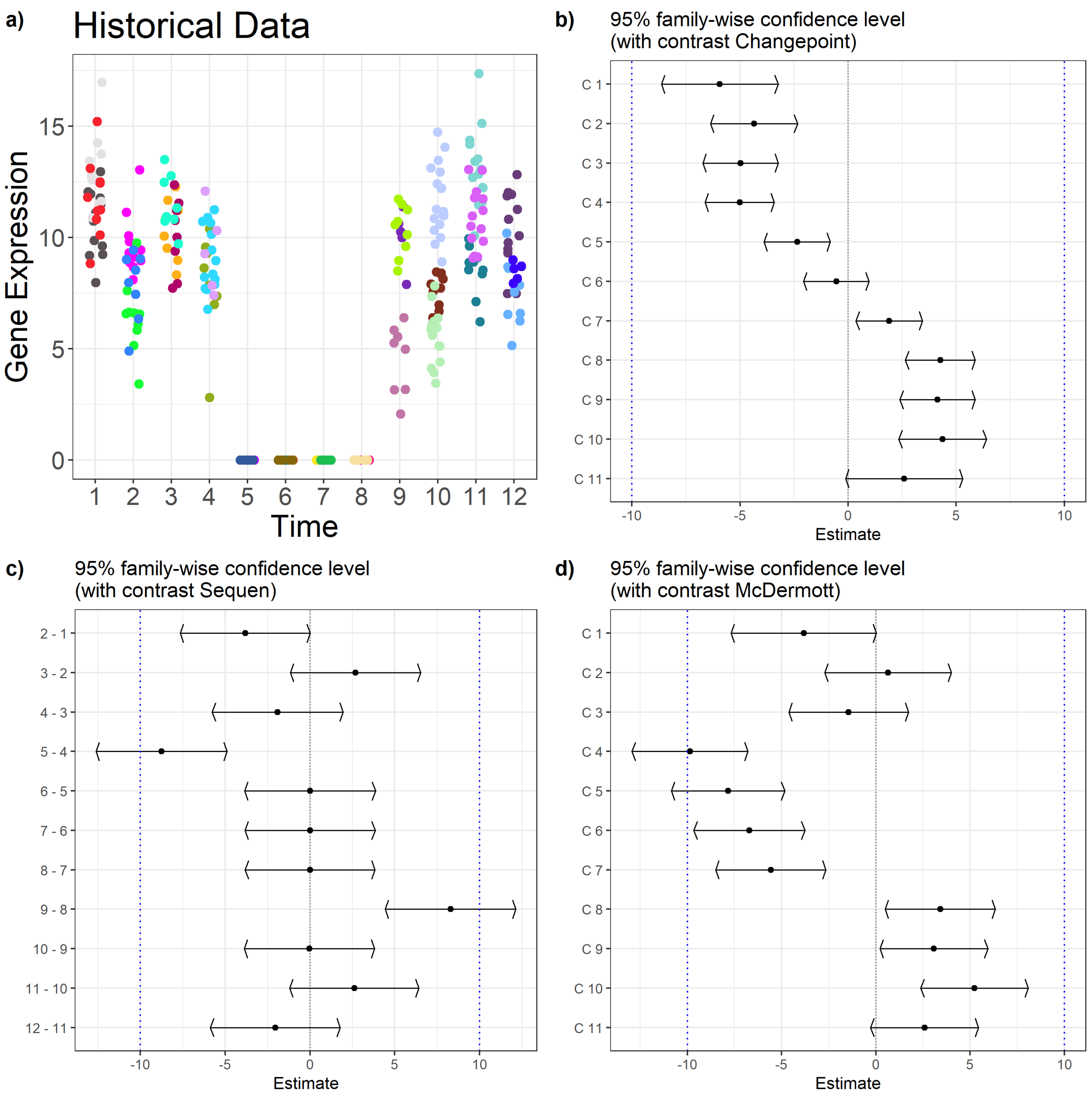

Supplementary Figure 13: **Confidence intervals of estimates from linear mixed model coupled with contrast matrix for historical data with a drop to total zero with zero variance.** Figure a) shows the increasing points in time (x-axis) of the sampled historical data in association with gene expression activity (y-axis) with no expected change point. Each color is related to one mother mouse. Subfigures b), c) and d) show the estimates (x-axis) including confidence intervals for the observed contrasts (y-axis) with methods Changepoint, Sequen and McDermott, respectively.

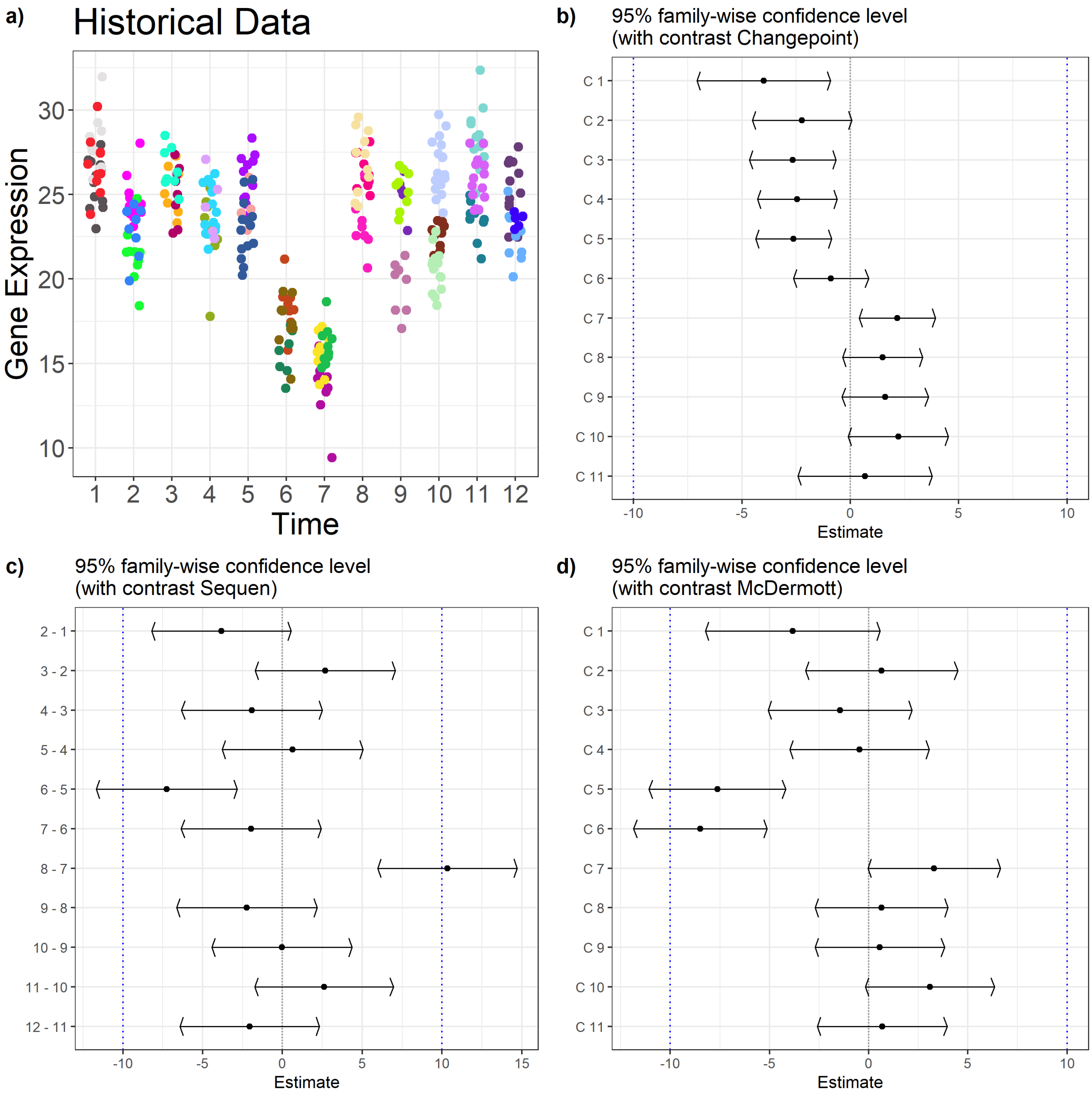

Supplementary Figure 14: **Confidence intervals of estimates from linear mixed model coupled with contrast matrix for historical data with a drop with variance.** Figure a) shows the increasing points in time (x-axis) of the sampled historical data in association with gene expression activity (y-axis) with no expected change point. Each color is related to one mother mouse. Subfigures b), c) and d) show the estimates (x-axis) including confidence intervals for the observed contrasts (y-axis) with methods Changepoint, Sequen and McDermott, respectively.

3.2 Additional Tables

Supplementary Table 4: Contrasts and estimates to supplementary figure 4. The table shows the numeric values from the simulation. The C column indicates the contrast, the Δ the log mean change of the corresponding contrast C. No change point was simulated. A significant confidence interval does not include zero.

Changepoint				Sequen				McDermott			
		95% CI				95% CI				95% CI	
C†	Δ‡	Low	Upp	C†	Δ‡	Low	Upp	C†	Δ‡	Low	Upp
C 1	-2.22	-5.31	0.86	2 - 1	-3.82	-8.19	0.55	C 1	-3.82	-8.23	0.58
C 2	-0.28	-2.58	2.02	3 - 2	2.68	-1.71	7.07	C 2	0.65	-3.19	4.49
C 3	-0.54	-2.54	1.45	4 - 3	-1.91	-6.32	2.50	C 3	-1.44	-5.07	2.19
C 4	-0.10	-1.93	1.73	5 - 4	0.63	-3.78	5.05	C 4	-0.47	-3.98	3.05
C 5	0.04	-1.71	1.80	6 - 5	2.74	-1.67	7.15	C 5	2.37	-1.07	5.81
C 6	-0.59	-2.32	1.14	7 - 6	-1.96	-6.34	2.42	C 6	0.07	-3.29	3.43
C 7	-0.74	-2.50	1.02	8 - 7	0.34	-4.02	4.71	C 7	0.40	-2.94	3.74
C 8	-1.04	-2.89	0.81	9 - 8	-2.23	-6.64	2.17	C 8	-1.89	-5.24	1.46
C 9	-0.70	-2.69	1.29	10 - 9	-0.03	-4.41	4.35	C 9	-1.75	-5.02	1.51
C 10	0.24	-2.07	2.55	11 - 10	2.61	-1.73	6.95	C 10	1.11	-2.15	4.37
C 11	-1.07	-4.17	2.03	12 - 11	-2.05	-6.42	2.32	C 11	-1.07	-4.34	2.21

† Given contrast.

‡ Point estimator of the confidence interval i.e. mean difference given the contrast.

Supplementary Table 5: Contrasts and estimates to supplementary figure 5. The table shows the numeric values from the simulation. The C column indicates the contrast, the Δ the log mean change of the corresponding contrast C. No change point was simulated. A significant confidence interval does not include zero.

Changepoint				Sequen				McDermott			
		95% CI				95% CI				95% CI	
C†	Δ‡	Low	Upp	C†	Δ‡	Low	Upp	C†	Δ‡	Low	Upp
C 1	10.33	7.24	13.41	2 - 1	-1.82	-6.19	2.55	C 1	-1.82	-6.23	2.58
C 2	12.36	10.06	14.66	3 - 2	4.68	0.29	9.08	C 2	3.71	-0.12	7.55
C 3	12.08	10.09	14.08	4 - 3	0.09	-4.32	4.50	C 3	2.77	-0.87	6.40
C 4	12.44	10.61	14.27	5 - 4	2.63	-1.78	7.05	C 4	4.74	1.22	8.26
C 5	12.50	10.74	14.25	6 - 5	4.74	0.33	9.16	C 5	8.51	5.07	11.95
C 6	11.76	10.03	13.49	7 - 6	0.04	-4.35	4.42	C 6	7.32	3.96	10.68
C 7	11.46	9.69	13.22	8 - 7	2.34	-2.03	6.71	C 7	8.42	5.08	11.76
C 8	11.06	9.22	12.91	9 - 8	-0.23	-4.64	4.17	C 8	7.12	3.77	10.47
C 9	11.24	9.25	13.23	10 - 9	1.97	-2.42	6.35	C 9	8.46	5.20	11.73
C 10	11.81	9.49	14.12	11 - 10	4.61	0.26	8.95	C 10	11.83	8.57	15.09
C 11	10.42	7.32	13.53	12 - 11	-0.05	-4.42	4.32	C 11	10.42	7.15	13.70

† Given contrast.

‡ Point estimator of the confidence interval i.e. mean difference given the contrast.

Supplementary Table 6: Contrasts and estimates to supplementary figure 6. The table shows the numeric values from the simulation. The C column indicates the contrast, the Δ the log mean change of the corresponding contrast C. The gray row indicates the predefined change point(s). A significant confidence interval does not include zero.

Changepoint				Sequen				McDermott			
		95% CI				95% CI				95% CI	
C†	Δ‡	Low	Upp	C†	Δ‡	Low	Upp	C†	Δ‡	Low	Upp
C 1	3.71	0.62	6.79	2 - 1	-3.82	-8.19	0.54	C 1	-3.82	-8.23	0.58
C 2	6.23	3.93	8.53	3 - 2	2.68	-1.71	7.07	C 2	0.65	-3.19	4.49
C 3	6.54	4.54	8.53	4 - 3	-1.91	-6.32	2.50	C 3	-1.44	-5.07	2.19
C 4	7.75	5.92	9.59	5 - 4	0.63	-3.78	5.05	C 4	-0.47	-3.98	3.05
C 5	8.94	7.19	10.70	6 - 5	2.74	-1.67	7.15	C 5	2.37	-1.07	5.81
C 6	9.41	7.68	11.15	7 - 6	8.04	3.66	12.42	C 6	10.07	6.71	13.43
C 7	7.56	5.80	9.33	8 - 7	0.34	-4.02	4.71	C 7	8.70	5.36	12.04
C 8	6.21	4.36	8.06	9 - 8	-2.23	-6.64	2.17	C 8	5.36	2.01	8.71
C 9	5.91	3.92	7.90	10 - 9	-0.03	-4.41	4.35	C 9	4.86	1.60	8.12
C 10	5.89	3.57	8.20	11 - 10	2.61	-1.73	6.94	C 10	6.76	3.50	10.01
C 11	3.93	0.83	7.03	12 - 11	-2.05	-6.42	2.32	C 11	3.93	0.66	7.21

† Given contrast.

‡ Point estimator of the confidence interval i.e. mean difference given the contrast.

Supplementary Table 7: Contrasts and estimates to supplementary figure 7. The table shows the numeric values from the simulation. The C column indicates the contrast, the Δ the log mean change of the corresponding contrast C. The gray row indicates the predefined change point(s). A significant confidence interval does not include zero.

Changepoint				Sequen				McDermott			
		95% CI				95% CI				95% CI	
C†	Δ‡	Low	Upp	C†	Δ‡	Low	Upp	C†	Δ‡	Low	Upp
C 1	9.34	6.25	12.42	2 - 1	-3.82	-8.19	0.55	C 1	-3.82	-8.23	0.58
C 2	12.40	10.10	14.70	3 - 2	2.68	-1.71	7.08	C 2	0.65	-3.19	4.49
C 3	13.25	11.26	15.25	4 - 3	-1.91	-6.32	2.50	C 3	-1.44	-5.07	2.19
C 4	15.20	13.37	17.04	5 - 4	10.63	6.22	15.05	C 4	9.53	6.02	13.05
C 5	14.01	12.25	15.77	6 - 5	2.74	-1.67	7.15	C 5	10.33	6.89	13.77
C 6	12.97	11.24	14.70	7 - 6	-1.96	-6.34	2.42	C 6	6.87	3.51	10.24
C 7	13.10	11.34	14.86	8 - 7	0.34	-4.03	4.71	C 7	6.05	2.71	9.39
C 8	13.90	12.05	15.74	9 - 8	7.77	3.36	12.17	C 8	13.05	9.70	16.40
C 9	12.92	10.93	14.91	10 - 9	-0.03	-4.42	4.35	C 9	11.87	8.61	15.14
C 10	11.87	9.56	14.18	11 - 10	2.61	-1.73	6.95	C 10	12.74	9.49	16.00
C 11	9.23	6.13	12.34	12 - 11	-2.05	-6.42	2.32	C 11	9.23	5.96	12.51

† Given contrast.

‡ Point estimator of the confidence interval i.e. mean difference given the contrast.

Supplementary Table 8: Contrasts and estimates to supplementary figure 8. The table shows the numeric values from the simulation. The C column indicates the contrast, the Δ the log mean change of the corresponding contrast C. The gray row indicates the predefined change point(s). A significant confidence interval does not include zero.

Changepoint				Sequen				McDermott			
		95% CI				95% CI				95% CI	
C†	Δ‡	Low	Upp	C†	Δ‡	Low	Upp	C†	Δ‡	Low	Upp
C 1	15.42	12.34	18.50	2 - 1	-3.82	-8.19	0.54	C 1	-3.82	-8.23	0.58
C 2	19.08	16.78	21.38	3 - 2	2.68	-1.71	7.07	C 2	0.65	-3.19	4.49
C 3	20.52	18.52	22.51	4 - 3	8.09	3.68	12.50	C 3	8.56	4.93	12.19
C 4	19.78	17.94	21.61	5 - 4	0.63	-3.78	5.05	C 4	7.15	3.63	10.66
C 5	20.00	18.24	21.76	6 - 5	2.74	-1.67	7.15	C 5	8.43	4.99	11.87
C 6	20.21	18.48	21.94	7 - 6	8.04	3.66	12.42	C 6	15.25	11.89	18.61
C 7	18.68	16.91	20.44	8 - 7	0.34	-4.02	4.71	C 7	13.00	9.66	16.34
C 8	18.29	16.44	20.13	9 - 8	-2.23	-6.64	2.17	C 8	9.12	5.77	12.47
C 9	19.34	17.35	21.33	10 - 9	9.97	5.59	14.35	C 9	18.29	15.02	21.55
C 10	17.35	15.03	19.66	11 - 10	2.61	-1.73	6.94	C 10	18.22	14.96	21.48
C 11	14.08	10.98	17.19	12 - 11	-2.05	-6.42	2.32	C 11	14.08	10.81	17.36

† Given contrast.

‡ Point estimator of the confidence interval i.e. mean difference given the contrast.

Supplementary Table 9: Contrasts and estimates to supplementary figure 9. The table shows the numeric values from the simulation. The C column indicates the contrast, the Δ the log mean change of the corresponding contrast C. No change point was simulated. A significant confidence interval does not include zero.

Changepoint				Sequen				McDermott			
		95% CI				95% CI				95% CI	
C†	Δ‡	Low	Upp	C†	Δ‡	Low	Upp	C†	Δ‡	Low	Upp
C 1	-14.77	-17.86	-11.69	2 - 1	-5.82	-10.19	-1.46	C 1	-5.82	-10.23	-1.42
C 2	-12.92	-15.22	-10.62	3 - 2	0.68	-3.71	5.07	C 2	-2.42	-6.25	1.42
C 3	-13.17	-15.16	-11.17	4 - 3	-3.91	-8.32	0.50	C 3	-5.65	-9.28	-2.02
C 4	-12.64	-14.47	-10.81	5 - 4	-1.37	-5.78	3.05	C 4	-5.67	-9.19	-2.15
C 5	-12.41	-14.17	-10.65	6 - 5	0.74	-3.67	5.15	C 5	-3.77	-7.21	-0.33
C 6	-12.93	-14.67	-11.20	7 - 6	-3.96	-8.34	0.42	C 6	-7.19	-10.55	-3.82
C 7	-12.93	-14.70	-11.17	8 - 7	-1.66	-6.02	2.71	C 7	-7.62	-10.96	-4.28
C 8	-13.14	-14.99	-11.29	9 - 8	-4.23	-8.64	0.17	C 8	-10.89	-14.24	-7.54
C 9	-12.64	-14.63	-10.65	10 - 9	-2.03	-6.41	2.35	C 9	-11.97	-15.23	-8.71
C 10	-11.33	-13.64	-9.02	11 - 10	0.61	-3.73	4.94	C 10	-9.61	-12.87	-6.35
C 11	-12.56	-15.67	-9.46	12 - 11	-4.05	-8.42	0.32	C 11	-12.56	-15.84	-9.29

† Given contrast.

‡ Point estimator of the confidence interval i.e. mean difference given the contrast.

Supplementary Table 10: Contrasts and estimates to supplementary figure 10. The table shows the numeric values from the simulation. The C column indicates the contrast, the Δ the log mean change of the corresponding contrast C. The gray row indicates the predefined change point(s). A significant confidence interval does not include zero.

Changepoint				Sequen				McDermott			
		95% CI				95% CI				95% CI	
C†	Δ‡	Low	Upp	C†	Δ‡	Low	Upp	C†	Δ‡	Low	Upp
C 1	-8.16	-11.24	-5.07	2 - 1	-3.82	-8.19	0.54	C 1	-3.82	-8.23	0.58
C 2	-6.79	-9.09	-4.49	3 - 2	2.68	-1.71	7.07	C 2	0.65	-3.19	4.49
C 3	-7.62	-9.62	-5.63	4 - 3	-1.91	-6.32	2.50	C 3	-1.44	-5.07	2.19
C 4	-7.95	-9.79	-6.12	5 - 4	0.63	-3.78	5.05	C 4	-0.47	-3.98	3.05
C 5	-8.86	-10.61	-7.10	6 - 5	2.74	-1.67	7.15	C 5	2.37	-1.07	5.81
C 6	-10.59	-12.32	-8.85	7 - 6	-11.96	-16.34	-7.58	C 6	-9.93	-13.29	-6.57
C 7	-9.04	-10.80	-7.27	8 - 7	0.34	-4.02	4.71	C 7	-7.90	-11.24	-4.56
C 8	-8.29	-10.14	-6.44	9 - 8	-2.23	-6.64	2.17	C 8	-9.14	-12.48	-5.79
C 9	-7.32	-9.31	-5.33	10 - 9	-0.03	-4.41	4.35	C 9	-8.37	-11.63	-5.10
C 10	-5.41	-7.72	-3.09	11 - 10	2.61	-1.73	6.94	C 10	-4.54	-7.79	-1.28
C 11	-6.07	-9.17	-2.97	12 - 11	-2.05	-6.42	2.32	C 11	-6.07	-9.34	-2.79

† Given contrast.

‡ Point estimator of the confidence interval i.e. mean difference given the contrast.

Supplementary Table 11: Contrasts and estimates to supplementary figure 11. The table shows the numeric values from the simulation. The C column indicates the contrast, the Δ the log mean change of the corresponding contrast C. The gray row indicates the predefined change point(s). A significant confidence interval does not include zero.

Changepoint				Sequen				McDermott			
		95% CI				95% CI				95% CI	
C†	Δ‡	Low	Upp	C†	Δ‡	Low	Upp	C†	Δ‡	Low	Upp
C 1	-13.78	-16.87	-10.70	2 - 1	-3.82	-8.19	0.55	C 1	-3.82	-8.23	0.58
C 2	-12.97	-15.26	-10.67	3 - 2	2.68	-1.71	7.08	C 2	0.65	-3.19	4.49
C 3	-14.34	-16.33	-12.35	4 - 3	-1.91	-6.32	2.50	C 3	-1.44	-5.07	2.19
C 4	-15.40	-17.24	-13.57	5 - 4	-9.37	-13.78	-4.95	C 4	-10.47	-13.98	-6.95
C 5	-13.92	-15.68	-12.17	6 - 5	2.74	-1.67	7.16	C 5	-5.59	-9.03	-2.14
C 6	-14.15	-15.88	-12.41	7 - 6	-1.96	-6.35	2.42	C 6	-6.74	-10.10	-3.38
C 7	-14.57	-16.34	-12.81	8 - 7	0.34	-4.03	4.71	C 7	-5.25	-8.59	-1.91
C 8	-15.97	-17.82	-14.12	9 - 8	-12.23	-16.64	-7.83	C 8	-16.82	-20.17	-13.47
C 9	-14.33	-16.32	-12.34	10 - 9	-0.03	-4.42	4.35	C 9	-15.38	-18.64	-12.12
C 10	-11.39	-13.71	-9.08	11 - 10	2.61	-1.74	6.95	C 10	-10.52	-13.78	-7.27
C 11	-11.37	-14.47	-8.27	12 - 11	-2.05	-6.42	2.32	C 11	-11.37	-14.65	-8.10

† Given contrast.

‡ Point estimator of the confidence interval i.e. mean difference given the contrast.

Supplementary Table 12: Contrasts and estimates to supplementary figure 12. The table shows the numeric values from the simulation. The C column indicates the contrast, the Δ the log mean change of the corresponding contrast C. The gray row indicates the predefined change point(s). A significant confidence interval does not include zero.

Changepoint				Sequen				McDermott			
		95% CI				95% CI				95% CI	
C†	Δ‡	Low	Upp	C†	Δ‡	Low	Upp	C†	Δ‡	Low	Upp
C 1	-19.87	-22.95	-16.79	2 - 1	-3.82	-8.20	0.55	C 1	-3.82	-8.23	0.58
C 2	-19.64	-21.94	-17.34	3 - 2	2.68	-1.71	7.08	C 2	0.65	-3.19	4.49
C 3	-21.60	-23.60	-19.61	4 - 3	-11.91	-16.32	-7.50	C 3	-11.44	-15.07	-7.81
C 4	-19.98	-21.81	-18.14	5 - 4	0.63	-3.79	5.05	C 4	-8.08	-11.60	-4.56
C 5	-19.91	-21.67	-18.15	6 - 5	2.74	-1.67	7.16	C 5	-3.69	-7.13	-0.24
C 6	-21.39	-23.12	-19.65	7 - 6	-11.96	-16.35	-7.58	C 6	-15.11	-18.48	-11.75
C 7	-20.15	-21.91	-18.39	8 - 7	0.34	-4.03	4.71	C 7	-12.20	-15.54	-8.86
C 8	-20.36	-22.21	-18.51	9 - 8	-2.23	-6.64	2.18	C 8	-12.89	-16.24	-9.54
C 9	-20.74	-22.73	-18.75	10 - 9	-10.03	-14.42	-5.65	C 9	-21.79	-25.06	-18.53
C 10	-16.87	-19.18	-14.56	11 - 10	2.61	-1.74	6.95	C 10	-16.00	-19.26	-12.74
C 11	-16.22	-19.32	-13.12	12 - 11	-2.05	-6.43	2.32	C 11	-16.22	-19.49	-12.95

† Given contrast.
‡ Point estimator of the confidence interval i.e. mean difference given the contrast.

Supplementary Table 13: Contrasts and estimates to supplementary figure 13. The table shows the numeric values from the simulation. The C column indicates the contrast, the Δ the log mean change of the corresponding contrast C. The gray row indicates the predefined change point(s). A significant confidence interval does not include zero.

Changepoint				Sequen				McDermott			
		95% CI				95% CI				95% CI	
C†	Δ‡	Low	Upp	C†	Δ‡	Low	Upp	C†	Δ‡	Low	Upp
C 1	-5.94	-8.64	-3.24	2 - 1	-3.82	-7.65	0.00	C 1	-3.82	-7.68	0.03
C 2	-4.36	-6.37	-2.34	3 - 2	2.68	-1.16	6.53	C 2	0.65	-2.71	4.01
C 3	-4.98	-6.72	-3.23	4 - 3	-1.91	-5.77	1.95	C 3	-1.44	-4.62	1.73
C 4	-5.02	-6.62	-3.41	5 - 4	-8.76	-12.62	-4.90	C 4	-9.86	-12.94	-6.78
C 5	-2.36	-3.90	-0.82	6 - 5	0.00	-3.86	3.86	C 5	-7.84	-10.86	-4.83
C 6	-0.55	-2.06	0.97	7 - 6	0.00	-3.84	3.84	C 6	-6.71	-9.65	-3.77
C 7	1.90	0.36	3.45	8 - 7	0.00	-3.82	3.82	C 7	-5.57	-8.49	-2.65
C 8	4.26	2.64	5.88	9 - 8	8.28	4.43	12.14	C 8	3.42	0.49	6.35
C 9	4.13	2.39	5.88	10 - 9	-0.04	-3.87	3.80	C 9	3.08	0.22	5.94
C 10	4.37	2.34	6.39	11 - 10	2.61	-1.20	6.41	C 10	5.24	2.39	8.09
C 11	2.59	-0.13	5.30	12 - 11	-2.05	-5.88	1.77	C 11	2.59	-0.28	5.45

† Given contrast.
‡ Point estimator of the confidence interval i.e. mean difference given the contrast.

Supplementary Table 14: Contrasts and estimates to supplementary figure 14. The table shows the numeric values from the simulation. The C column indicates the contrast, the Δ the log mean change of the corresponding contrast C. The gray row indicates the predefined change point(s). A significant confidence interval does not include zero.

Changepoint				Sequen				McDermott			
		95% CI				95% CI				95% CI	
C†	Δ‡	Low	Upp	C†	Δ‡	Low	Upp	C†	Δ‡	Low	Upp
C 1	-4.00	-7.08	-0.92	2 - 1	-3.82	-8.19	0.55	C 1	-3.82	-8.23	0.58
C 2	-2.23	-4.52	0.07	3 - 2	2.68	-1.71	7.07	C 2	0.65	-3.19	4.49
C 3	-2.66	-4.65	-0.67	4 - 3	-1.91	-6.32	2.50	C 3	-1.44	-5.07	2.19
C 4	-2.45	-4.28	-0.62	5 - 4	0.63	-3.78	5.05	C 4	-0.47	-3.98	3.05
C 5	-2.62	-4.37	-0.86	6 - 5	-7.26	-11.67	-2.85	C 5	-7.63	-11.07	-4.19
C 6	-0.89	-2.62	0.84	7 - 6	-1.96	-6.34	2.42	C 6	-8.49	-11.85	-5.13
C 7	2.16	0.40	3.92	8 - 7	10.34	5.98	14.71	C 7	3.30	-0.04	6.64
C 8	1.49	-0.35	3.34	9 - 8	-2.23	-6.64	2.17	C 8	0.65	-2.70	4.00
C 9	1.61	-0.38	3.60	10 - 9	-0.03	-4.41	4.35	C 9	0.56	-2.71	3.82
C 10	2.21	-0.10	4.52	11 - 10	2.61	-1.73	6.95	C 10	3.08	-0.17	6.34
C 11	0.68	-2.42	3.78	12 - 11	-2.05	-6.42	2.32	C 11	0.68	-2.60	3.95

† Given contrast.

‡ Point estimator of the confidence interval i.e. mean difference given the contrast.

4 Analysis in R

An general tutorial on linear mixed models using contrasts in R can be found Schad et al. (2020) [2]. Also Bretz et al. (2011) [3] and Hothorn et al. (2008) [4] deliver the theoretical background in the context of multiple testing. If the code section is hard to read or you want a direct copy, please also refer to the GitHub repository for direct access to the R code: https://github.com/msieg08/clustered_data_changepoint_detection

```
pacman::p_load(MASS, plyr, magrittr, knitr, tidyverse, simstudy,
               multcomp, lme4, effects, pals, ggpubr, broom)

#### Functions ####
##' generate expression data per timepoint
generate_timepoint <- function(i, intercept = 50, ngen = 3){

  # generate mothers, mother effect and respective number of pups per timepoint
  gen.mother <- defData(varname = "mother", dist = "normal", formula = 0,
                        variance = 5, id = "idMother")
  gen.mother <- defData(gen.mother, varname = "nPups", dist = "noZeroPoisson",
                        ula = 10)
  dtMother <- genData(ngen, gen.mother)

  # generate expression data for all pups at timepoint with
  dtPups <- genCluster(dtMother, cLevelVar = "idMother", numIndsVar = "nPups",
                       level1ID = "idPups")
  gen.pup <- defDataAdd(varname = "gender", dist = "binary",
                        formula = 0.5)
  gen.pup <- defDataAdd(gen.pup, varname = "expression", dist = "normal",
                        formula = str_c(intercept, " + mother"),
                        variance = 2)

  dtMice <- addColumns(gen.pup, dtPups) %>% as_tibble()

  # set intercept of zero to have no variance; all expression values to zero
  if(intercept == 0){
    dtMice$expression <- 0
  }

  # clean data set
  dtMice <- dtMice %>%
    select(expression, idMother, gender, group_id = idPups, efMother = mother) %>%
    mutate(idMother = (idMother + ngen*i - ngen), timepoint = i)

  return(dtMice)
}
```

Code for figure (3) and table (3)

```
#### Analysis ####
fileName = "time_course_example_"
example_intercepts <- c(rep(25, 6), rep(35, 6))

# generate expression data for all timepoints in this scenario
set.seed(1308)
lsMice <- llply(1:length(example_intercepts), function(i)
  generate_timepoint(i, example_intercepts[i], ngen = 3))
dtMice <- bind_rows(lsMice) %>%
  mutate(timepoint = as.factor(timepoint), idMother = as.factor(idMother))

## linear mixed-effects model with mean parametrization
lmer_fit <- lmer(expression ~ 0 + timepoint + (1 | idMother), dtMice)

#generalized linear hypothesis testing correction with Changepoint
changepoint_contrast <- contrMat(n = as.numeric(table(dtMice$timepoint)),
                                 type = "Changepoint")

glht_fitCP <- glht(lmer_fit, linfct = changepoint_contrast)


# multiple testing correction for Sequen (neigbhor comparison (-1,1))
sequen_contrast <- contrMat(n = as.numeric(table(dtMice$timepoint)),
                            type = "Sequen")

glht_fitS <- glht(lmer_fit, linfct = sequen_contrast)

# multiple testing correction for McDermott
mcdermott_contrast <- contrMat(n = as.numeric(table(dtMice$timepoint)),
                               type = "McDermott")

glht_fitMD <- glht(lmer_fit, linfct = mcdermott_contrast)


cp_df <- glht_fitCP %>% confint() %>% tidy()
md_df <- glht_fitMD %>% confint() %>% tidy()
seq_df <- glht_fitS %>% confint() %>% tidy()

conf_int_df <- bind_cols(cp_df, seq_df, md_df)
names(conf_int_df) <- cross2(colnames(cp_df), c("cp", "seq", "md")) \%>\%
  map_chr(paste, sep = ".", collapse = ".")
conf_int_df
```

```
#### Visualization####
# simulated data
p01 <-
  ggplot(dtMice) +
  geom_jitter(mapping = aes(x = timepoint, y = expression, colour = idMother),
              width = 0.2, size=2.5) +
  scale_colour_manual(values = unname(polychrome(36))) +
  labs(y = "Gene Expression Activity", x = "Time", title = "Short Time Series") +
  theme_bw() + theme(legend.position = "none", text = element_text(size=20))

# generalized linear hypothesis testing with Changepoint contrast matrix
cp <- confint(glht_fitCP) \%>\%
  tidy \%>\%
  ggplot(aes(x=reorder(fct_inorder(contrast), desc(fct_inorder(contrast))), y=estimate)) +
  geom_hline(yintercept=0, linetype="11", colour="grey60") +
  geom_segment(aes(xend=reorder(fct_inorder(contrast), desc(fct_inorder(contrast))),
                   y=conf.low, yend=conf.high), size=0.4,
               arrow=arrow(ends="both", length=unit(0.09, "inches"), angle=70)) +
  geom_point() +
  geom_hline(yintercept = c(-10,10), color = "blue", linetype = "dotted") +
  coord_flip() +
  theme_bw() +
  labs(y = "Estimate", x = "", title = "95\% family-wise confidence level\n(with contrast Changepoint)")


# generalized linear hypothesis testing with Sequen contrast matrix
sequen <- confint(glht_fitS) %>%
  tidy %>%
  ggplot(aes(x=reorder(fct_inorder(contrast), desc(fct_inorder(contrast))), y=estimate)) +
  geom_hline(yintercept=0, linetype="11", colour="grey60") +
  geom_segment(aes(xend=reorder(fct_inorder(contrast), desc(fct_inorder(contrast))), y=conf.low, yend=conf.high
               arrow=arrow(ends="both", length=unit(0.09, "inches"), angle=70)) +
  geom_point() +
  coord_flip() +
  geom_hline(yintercept = c(-10,10), color = "blue", linetype = "dotted") +
  theme_bw() +
  labs(y = "Estimate", x = "", title = "95% family-wise confidence level\n(with contrast Sequen)")


# generalized linear hypothesis testing with McDermott contrast matrix
mcd <- confint(glht_fitMD) %>%
  tidy %>%
```

```
  ggplot(aes(x=reorder(fct_inorder(contrast), desc(fct_inorder(contrast))), y=estimate)) +
  geom_hline(yintercept=0, linetype="11", colour="grey60") +
  geom_segment(aes(xend=reorder(fct_inorder(contrast), desc(fct_inorder(contrast))), y=conf.low, yend=conf.high
               arrow=arrow(ends="both", length=unit(0.09, "inches"), angle=70)) +
  geom_point() +
  coord_flip() +
  geom_hline(yintercept = c(-10,10), color = "blue", linetype = "dotted") +
  theme_bw() +
  labs(y = "Estimate", x = "", title = "95% family-wise confidence level\n(with contrast McDermott)")

model_pl <- ggarrange(p01, cp, sequen, mcd,
                      ncol=2, nrow=2,   legend="none",
                      labels = c("a)", "b)", "c)", "d)"))
model_pl
```

5 Effect of the Litter Variance

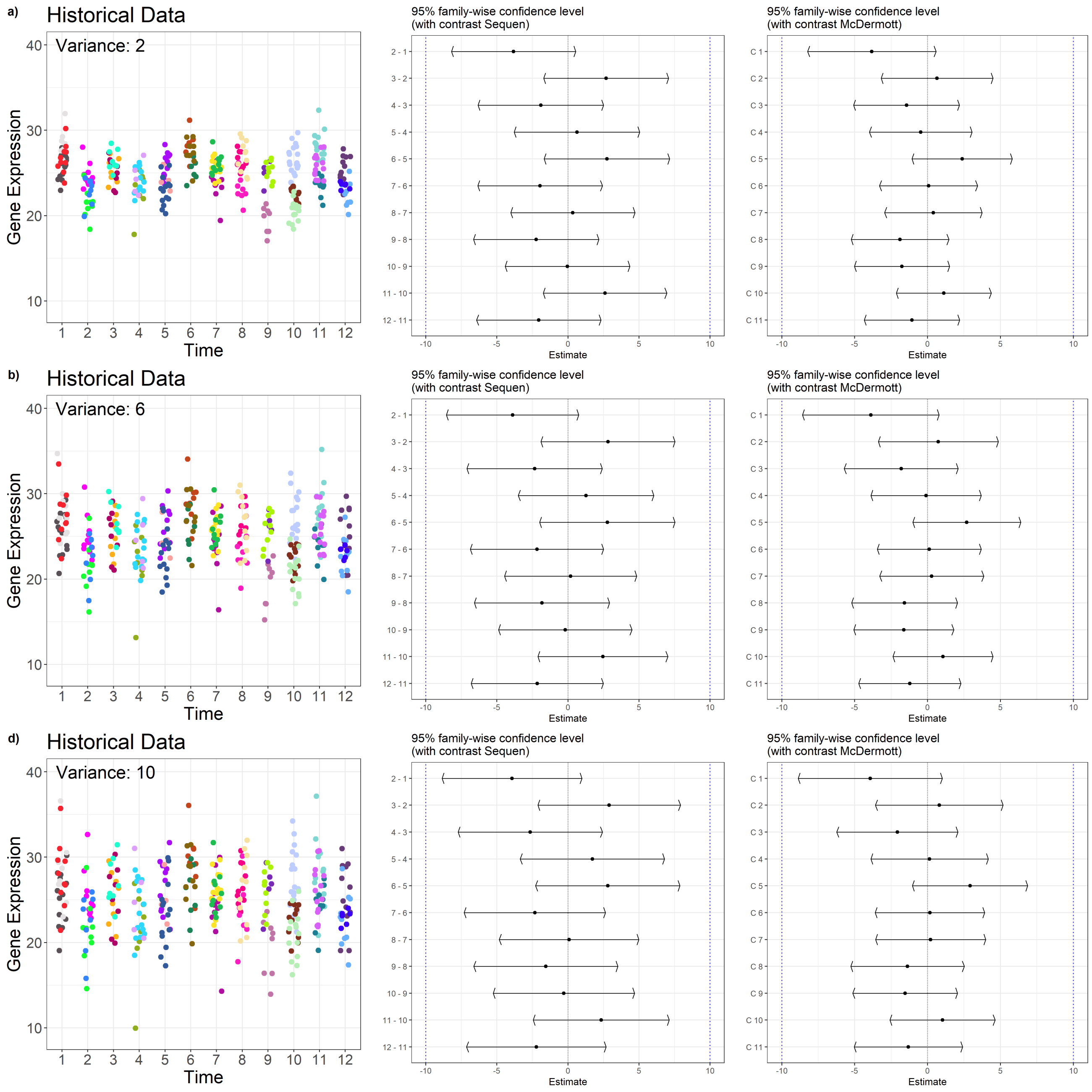

Supplementary Figure 15: **Effect of different litter variance on the course of the confidence intervals.** Figure row a) shows historical data with a variance of 2, figure row b) a variance of 6, and figure row c) a variance of 10. As can be seen, the different variants have no influence on the course of the Sequen and McDermott.

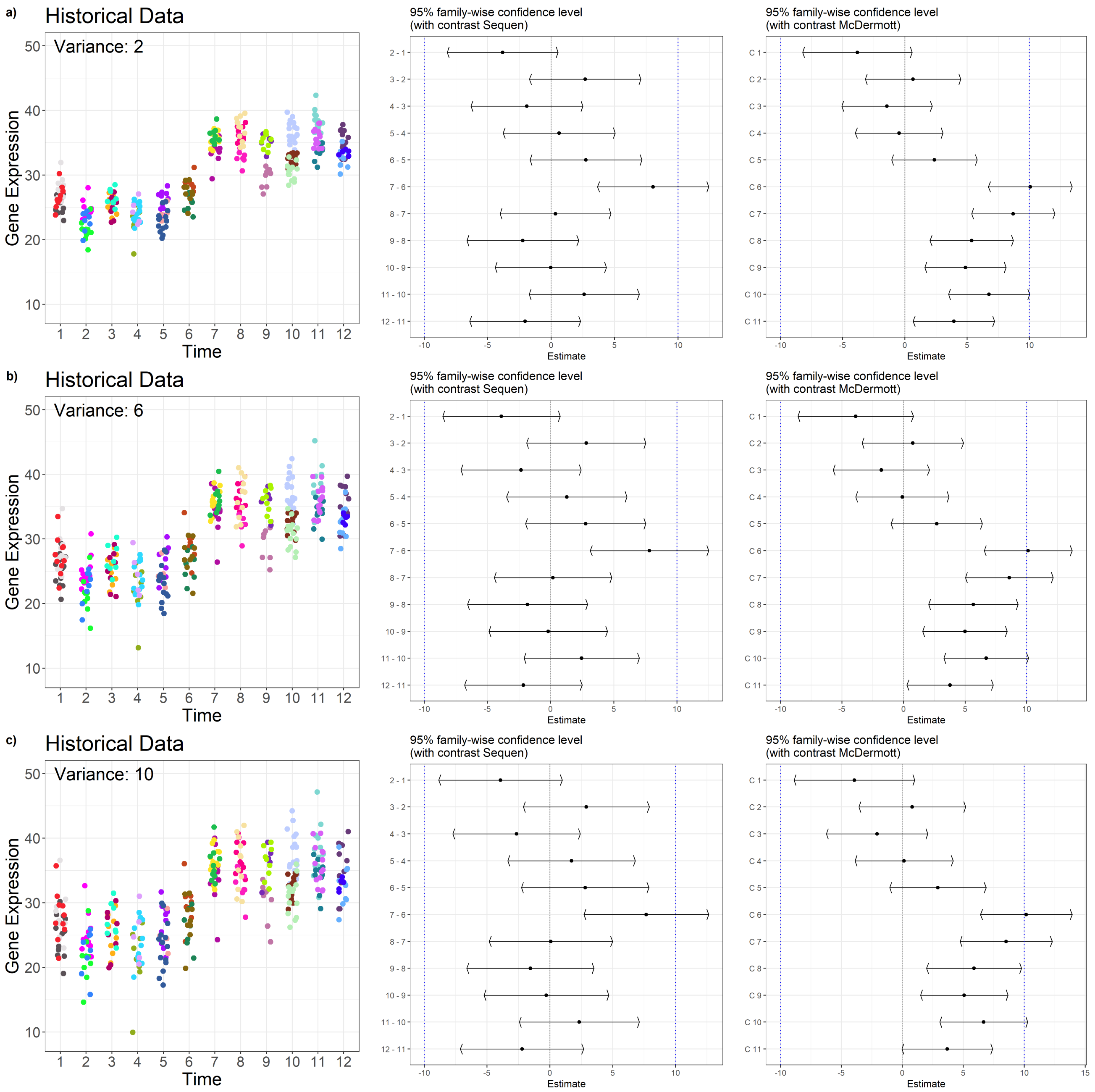

Supplementary Figure 16: **Effect of different litter variance on the course of the confidence intervals.** Figure row a) shows historical data with a variance of 2, figure row b) a variance of 6, and figure row c) a variance of 10. As can be seen, the different variants have no influence on the course of the Sequen and McDermott.

6 Flowchart of the Method

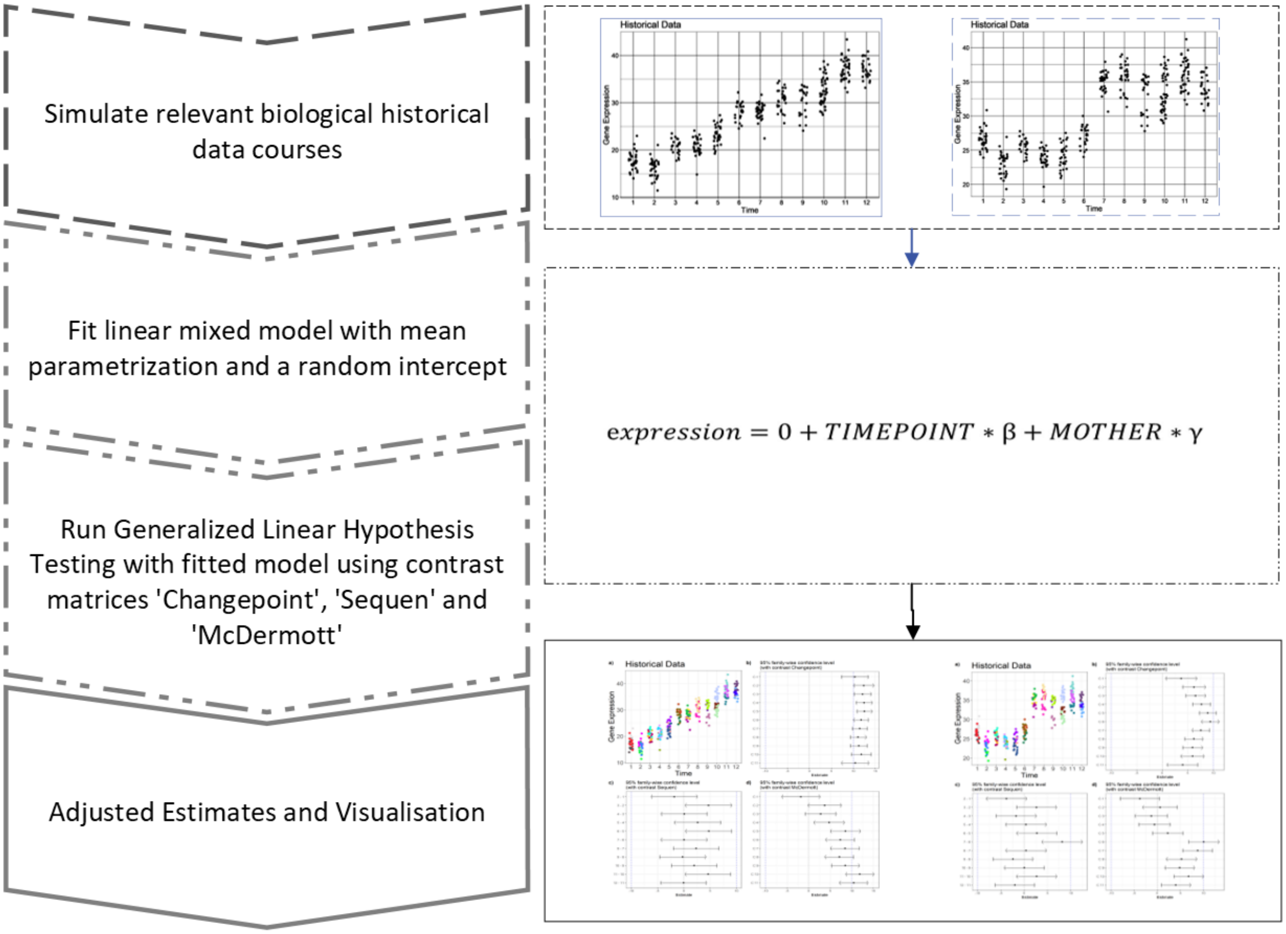

Supplementary Figure 17: **Flowchart of the methods from the simulation settings to the final figures** The corresponding R code to generate the final figures can be found in supplementary section 4. In addition see also the corresponding GitHub repository.

References

[1] Jochen Kruppa and Ludwig Hothorn. A comparison study on modeling of clustered and overdispersed count data for multiple comparisons. *Journal of Applied Statistics*, pages 1–13, 2020.

[2] Daniel J Schad, Shravan Vasishth, Sven Hohenstein, and Reinhold Kliegl. How to capitalize on a priori contrasts in linear (mixed) models: A tutorial. *Journal of Memory and Language*, 110:104038, 2020.

[3] Frank Bretz, Torsten Hothorn, and Peter Westfall. *Multiple comparisons using R*. CRC Press, Boca Raton, 2016.

[4] Torsten Hothorn, Frank Bretz, and Peter Westfall. Simultaneous inference in general parametric models. *Biometrical Journal: Journal of Mathematical Methods in Biosciences*, 50(3):346–363, 2008.
